# PAD4^+^ neutrophils promote hepatic stellate cell activation and accelerate MASH fibrosis progression viaNET-DNA/TAOK1/MAPK pathways

**DOI:** 10.1172/jci.insight.191479

**Published:** 2026-01-09

**Authors:** Jiajia Shen, Shanshan Huang, Yaohui Wang, Qingyuan Wang, Shibo Lin, Wei Guan, Yingyun Gong, Yiming Si, Ming Zhao, Hongwen Zhou, Hui Liang

**Affiliations:** 1 Department of General Surgery, First Affiliated Hospital, Nanjing Medical University, Nanjing, China.; 2 Department of Endocrinology, Endocrine and Metabolic Disease Medical Center, Nanjing Drum Tower Hospital, Affiliated Hospital of Medical School, Nanjing University, Nanjing, China.; 3 Branch of National Clinical Research Centre for Metabolic Diseases, Nanjing, China.; 4 Department of Pathology, Jiangsu Province Hospital of Chinese Medicine, Affiliated Hospital of Nanjing University of Chinese Medicine, Nanjing, China.; 5 Department of Endocrinology and Metabolism, First Affiliated Hospital, Nanjing Medical University, Nanjing, China.

**Keywords:** Cell biology, Hepatology, Metabolism, Fibrosis, Neutrophils

## Abstract

Neutrophils play a pivotal role in the progression of metabolic dysfunction–associated steatohepatitis (MASH) by mediating inflammatory responses. However, the heterogeneity of neutrophil subsets in MASH and their specific contributions to disease progression remain unclear. In this study, analysis of liver biopsies from 265 patients revealed a strong association between elevated neutrophil counts and MASH severity, particularly fibrosis. Five distinct neutrophil subsets were identified in human liver tissue, with PAD4^+^ neutrophils serving as key drivers in MASH progression. Mechanistically, PAD4^+^ neutrophils generate neutrophil extracellular traps (NETs) and activate hepatic stellate cells via the TAOK1-dependent MAPK signaling pathway. Inhibition of PAD4^+^ neutrophils in vivo attenuated the progression of liver fibrosis without exacerbating liver injury. Collectively, these findings elucidate the pivotal involvement of PAD4^+^ neutrophils in MASH progression and identify them as promising therapeutic targets for mitigating fibrosis and inflammation.

## Abstract 2

PAD4⁺ neutrophils drive liver fibrosis in MASH by releasing NETs that activate stellate cells via TAOK1–MAPK signaling, highlighting a potential therapeutic target.

## Graphical abstract

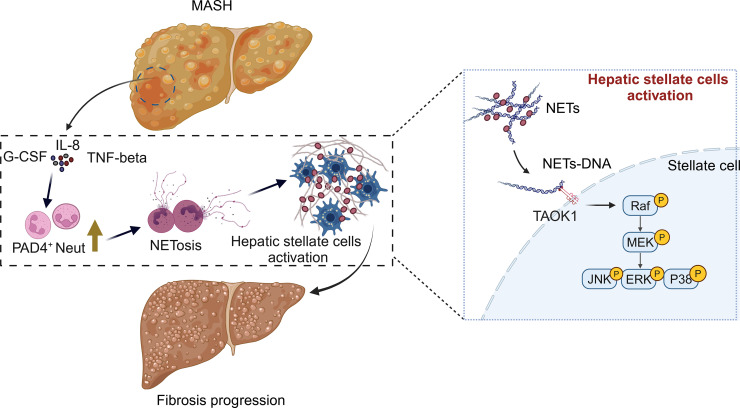

## Introduction

Metabolic dysfunction–associated steatotic liver disease (MASLD) has become the most common chronic liver disorder globally, affecting approximately 30% of adults (1). Metabolic dysfunction–associated steatohepatitis (MASH), an advanced stage of MASLD, is characterized by excessive fat accumulation, inflammation, hepatocellular injury, and fibrosis. It has received significant attention due to its potential progression to cirrhosis, or hepatocellular carcinoma (2, 3). Currently, there are limited therapeutic options for MASH, representing a significant unmet medical need (4).

Increasing evidence suggests that immune cell infiltration into the liver plays a crucial role in MASH, with inflammation being a major driver of disease pathology (5). Among immune cell types, neutrophils — key components of the innate immune response — are known to be involved in inflammation and tissue remodeling. Recent studies have highlighted an association between neutrophil infiltration and fibrosis severity in MASH, suggesting a possible link between neutrophils and disease progression (6, 7). However, the specific mechanisms by which neutrophils contribute to MASH pathogenesis remain poorly understood.

Neutrophils exhibit significant phenotypic and functional diversity (8, 9). In the steady state, single-cell RNA sequencing has identified 8 distinct populations of neutrophils, each characterized by unique molecular signatures (10). During bacterial infections, substantial changes occur in the genetic composition of neutrophil populations, resulting in dynamic transitions between subpopulations (10). In LPS-induced liver injury, transcriptomic analyses demonstrate a marked upregulation of the integrin subunit α_M_ in neutrophils compared with those isolated from healthy livers (11). In alcohol-associated hepatitis, low-density neutrophils display an exhausted phenotype with diminished phagocytic activity and originate from activated high-density neutrophils (12). However, the regulatory role of neutrophil heterogeneity in MASH development remains unclear.

In this study, we investigated the role of PAD4^+^ neutrophils in the progression of MASH, with particular emphasis on their involvement in hepatic stellate cell (HepSC) activation and liver fibrosis. By analyzing liver biopsy samples from 265 MASLD patients, we observed a significant correlation between neutrophil infiltration and disease severity, especially fibrosis. In murine models of MASH, neutrophil depletion mitigated both steatosis and fibrosis, but exacerbated liver injury. Additionally, we identified 5 distinct neutrophil subsets infiltrating the human liver, with PAD4^+^ neutrophils playing a central role in the promotion of MASH-associated liver fibrosis. Inhibition of PAD4^+^ neutrophils or neutrophil extracellular trap (NET) formation significantly attenuated fibrosis progression. Mechanistically, NETs released by PAD4^+^ neutrophils activated HepSCs through the TAOK1-dependent MAPK pathway. These findings enhance our understanding of neutrophil heterogeneity in MASH and highlight potential therapeutic targets for mitigating fibrosis in this disease.

## Results

### Neutrophil infiltration correlates with MASLD severity.

**Figure 1:**
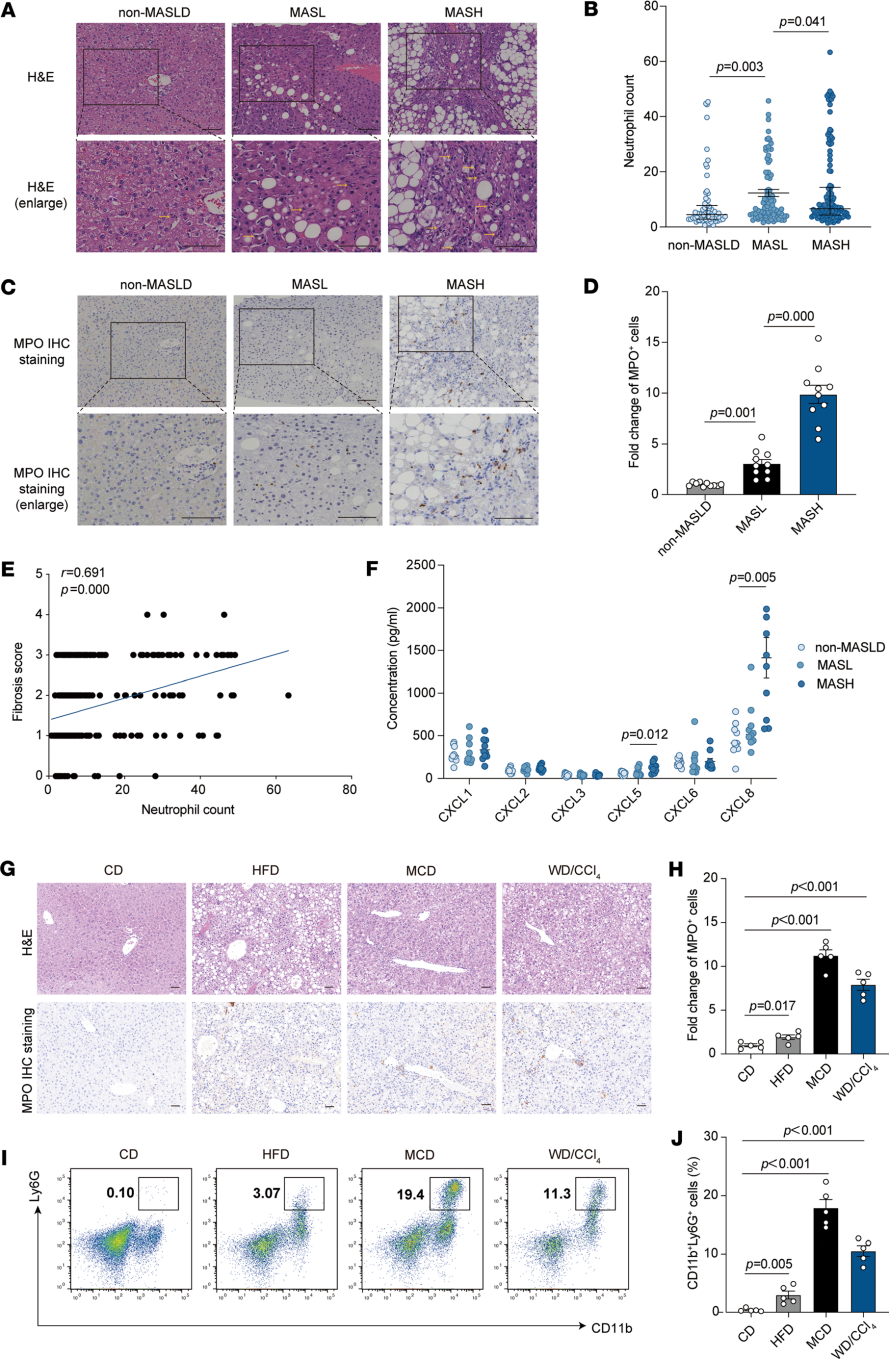
The presence of neutrophils in fatty liver tissue is proportionally linked to the severity of MASLD progression. (**A**) Hematoxylin and eosin (H&E) staining identified neutrophils in MASLD patient liver samples. These neutrophils were distinguishable by their segmented nuclei and granule-rich cytoplasm, exhibiting a reddish hue (indicated by yellow arrows). Scale bars: 100 μm. (**B**) The study involved comparing neutrophil levels in 3 patient groups: non-MASLD (*n* = 63), MASL (*n* = 77), and MASH (*n* = 125). Each sample underwent thorough examination by 2 experienced histologists, analyzing 6 high-power fields at ×400 magnification, each with a 0.55 μm field diameter. (**C**) Immunohistochemical (IHC) staining for MPO was used to identify neutrophils. Scale bars: 100 μm. (**D**) MPO^+^ cells’ staining intensity was quantified using ImageJ software (NIH). (**E**) Spearman’s bivariate correlation test was conducted to explore the relationship between neutrophil counts and fibrosis scores in a total of 265 cases. (**F**) ELISA was used to measure chemokine expression levels in human liver samples, comparing non-MASLD (*n* = 10), MASL (*n* = 10), and MASH (*n* = 10) patients. (**G**) IHC staining for MPO was used to identify neutrophils in the livers of mice fed with high-fat diet (HFD), methionine- and choline-deficient diet (MCD), and Western diet/carbon tetrachloride (WD/CCl_4_). Scale bars: 100 μm. (**H**) ImageJ software was used to evaluate MPO^+^ cell staining intensity in the livers of HFD-, MCD-, and WD/CCl_4_–fed mice. (**I** and **J**) Nonparenchymal cells (NPCs) were isolated from the livers of mice fed with HFD, MCD, and WD/CCl_4_. Flow cytometry assessed the levels of neutrophil infiltration (CD11b^+^Ly6G^+^) in CD45^+^ NPCs, comparing the results across these groups from 5 independent experiments. Statistical analyses were performed using 1-way ANOVA. Data are shown as the mean ± SEM.

To assess the clinical relevance of neutrophil infiltration in MASLD, we analyzed liver biopsies from patients with different disease severities. Neutrophil infiltration was significantly elevated in MASH compared with both metabolic dysfunction–associated steatotic liver (MASL) and non-MASLD groups, with neutrophils predominantly localized in the periportal regions and occasionally observed in the lobular areas (Figure 1, A and B). Immunohistochemical staining corroborated these findings, demonstrating an increased presence of neutrophils in the livers of MASH patients (Figure 1, C and D).

We assessed clinical and pathological characteristics in 265 liver biopsy samples, classifying them into non-MASLD (23.8%, *n* = 63), non-MASH (29.1%, *n* = 77), and MASH (47.1%, *n* = 125) cohorts (Supplemental Tables 1 and 2; supplemental material available online with this article; https://doi.org/10.1172/jci.insight.191479DS1). Patients with MASH were younger and exhibited worse metabolic profiles, including higher BMI and alanine aminotransferase (ALT), aspartate aminotransferase (AST), γ-glutamyltransferase (GGT), and total cholesterol (TC) levels (*P* < 0.05). Notably, neutrophil infiltration was significantly greater in the MASH group compared with both non-MASH and non-MASLD groups (*P* = 0.000; Supplemental Table 1). When clinicopathological data were stratified by tertiles of neutrophil counts, a positive correlation was observed between the degree of neutrophil infiltration and elevated levels of BMI, ALT, AST, GGT, and TC (*P* < 0.05). Additionally, neutrophil counts positively correlated with MASLD severity, including steatosis, ballooning, lobular inflammation, and fibrosis (*P* < 0.05; Table 1). Correlation analysis confirmed a strong association between neutrophil infiltration and liver fibrosis (*r* = 0.691) (Figure 1E and Table 2). We then assessed chemokine expression in human liver tissues using enzyme-linked immunosorbent assay and found elevated levels of CXCL5 and CXCL8 in the MASH group compared with both the non-MASLD and MASL groups (Figure 1F). Notably, CXCL8 was identified as a potent recruiter of neutrophils (13). Therefore, in human liver tissue, we observed a correlation between neutrophil infiltration and the pathological severity of MASLD.

To validate these findings, we established the MASL model by feeding mice a high-fat diet (HFD), and the MASH model by administering a methionine- and choline-deficient diet (MCD) or a Western diet combined with low-dose CCl_4_ (WD/CCl_4_). Neutrophil infiltration was significantly elevated in MASH mice compared with controls fed a standard diet, with the most pronounced infiltration observed in the MASH group (Figure 1, G and H). Flow cytometric analysis further confirmed these findings, revealing an increase in neutrophil populations in both MASL and MASH models (Figure 1, I and J). Collectively, these data suggest that neutrophil infiltration is a key feature of MASH, strongly correlating with disease severity, particularly fibrosis, and may serve as a potential clinical marker for disease progression.

### Neutrophil depletion inhibits the progression of hepatic steatosis and fibrosis but increases liver damage in MASH.

**Figure 2:**
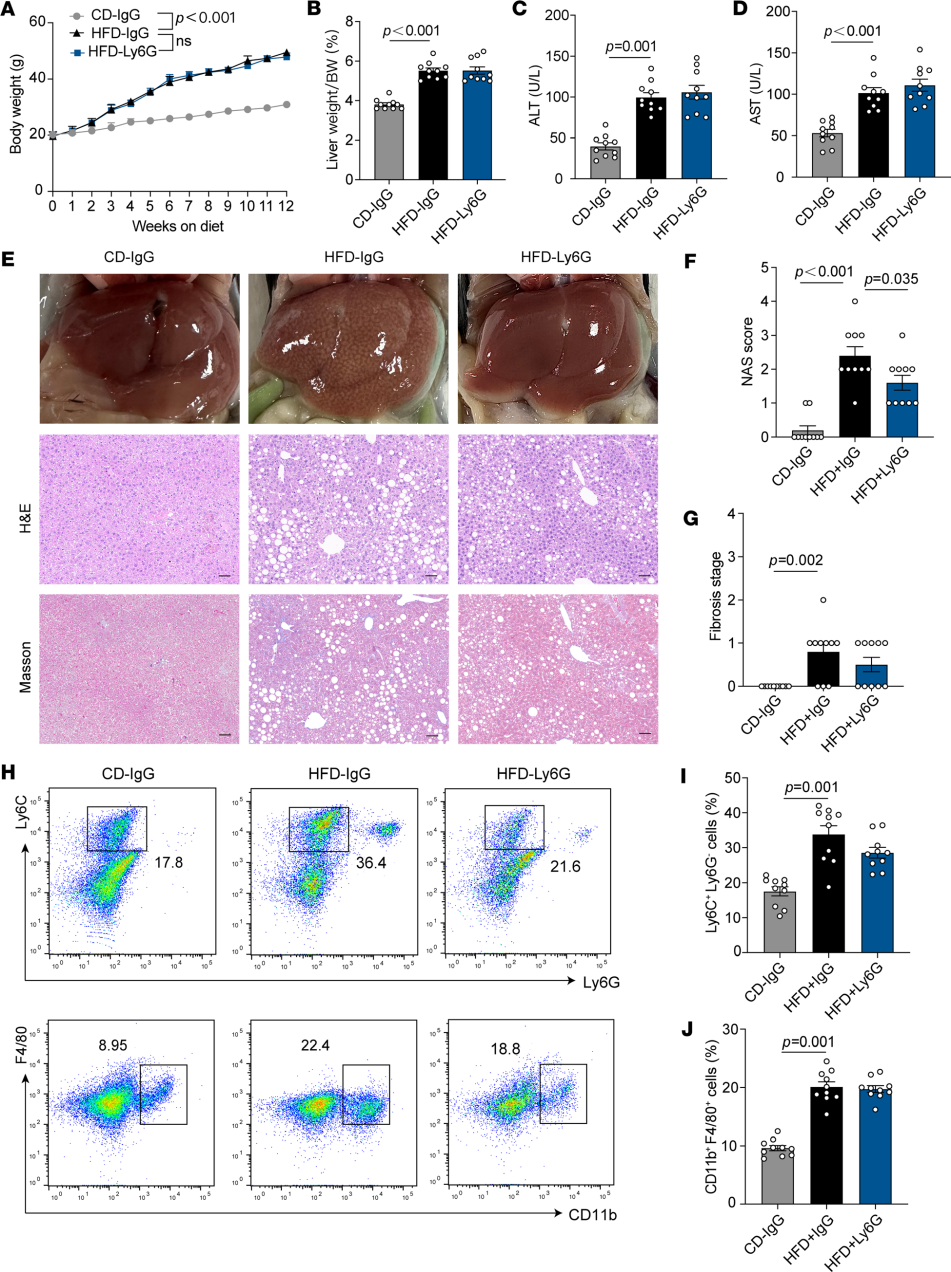
Effects of neutrophil depletion on liver under HFD model. (**A**) Weekly monitored body weight of CD-IgG–, HFD-IgG–, and HFD-Ly6G–treated mice (10 mice per group). (**B**) Liver weight/body weight ratio of mice in each group. (**C**) Serum ALT levels across different groups. (**D**) Serum AST levels across different groups. (**E**) Representative liver sections showing phenotypic changes, lipid accumulation, and fibrosis, assessed by Masson staining. Scale bars: 50 μm. (**F**) NAS scores of each group. (**G**) Fibrosis stage of each group. (**H**–**J**) Flow cytometry analysis of CD45^+^ NPCs extracted from HFD-fed mouse livers, showing monocyte (Ly6C^+^Ly6G^–^) and macrophage (CD11b^+^F4/80^+^) populations based on 10 independent experiments. Statistical analyses were performed using 1-way ANOVA. Data are shown as the mean ± SEM.

To investigate the effects of neutrophil depletion on liver pathology at the MASL and MASH stages, Ly6G antibodies were administered intraperitoneally during the development of the HFD and WD/CCl_4_ models (Supplemental Figure 1, A–C). In the HFD model, neutrophil depletion did not influence body weight gain (Figure 2A) or liver weight (Figure 2B). Serum levels of ALT and AST remained stable and were not significantly altered by neutrophil depletion (Figure 2, C and D). Notably, neutrophil depletion resulted in a reduction of the NAFLD activity score (NAS), primarily due to alleviated hepatic steatosis, while fibrosis levels exhibited only minor changes (Figure 2, E–G). Flow cytometric analysis revealed an increase in macrophage and monocyte infiltration into the liver under the HFD diet, although neutrophil depletion did not significantly affect this infiltration (Figure 2, H–J). Therefore, at the MASL stage, neutrophil clearance reduces hepatic steatosis while having minimal impact on inflammation and fibrosis.

**Figure 3:**
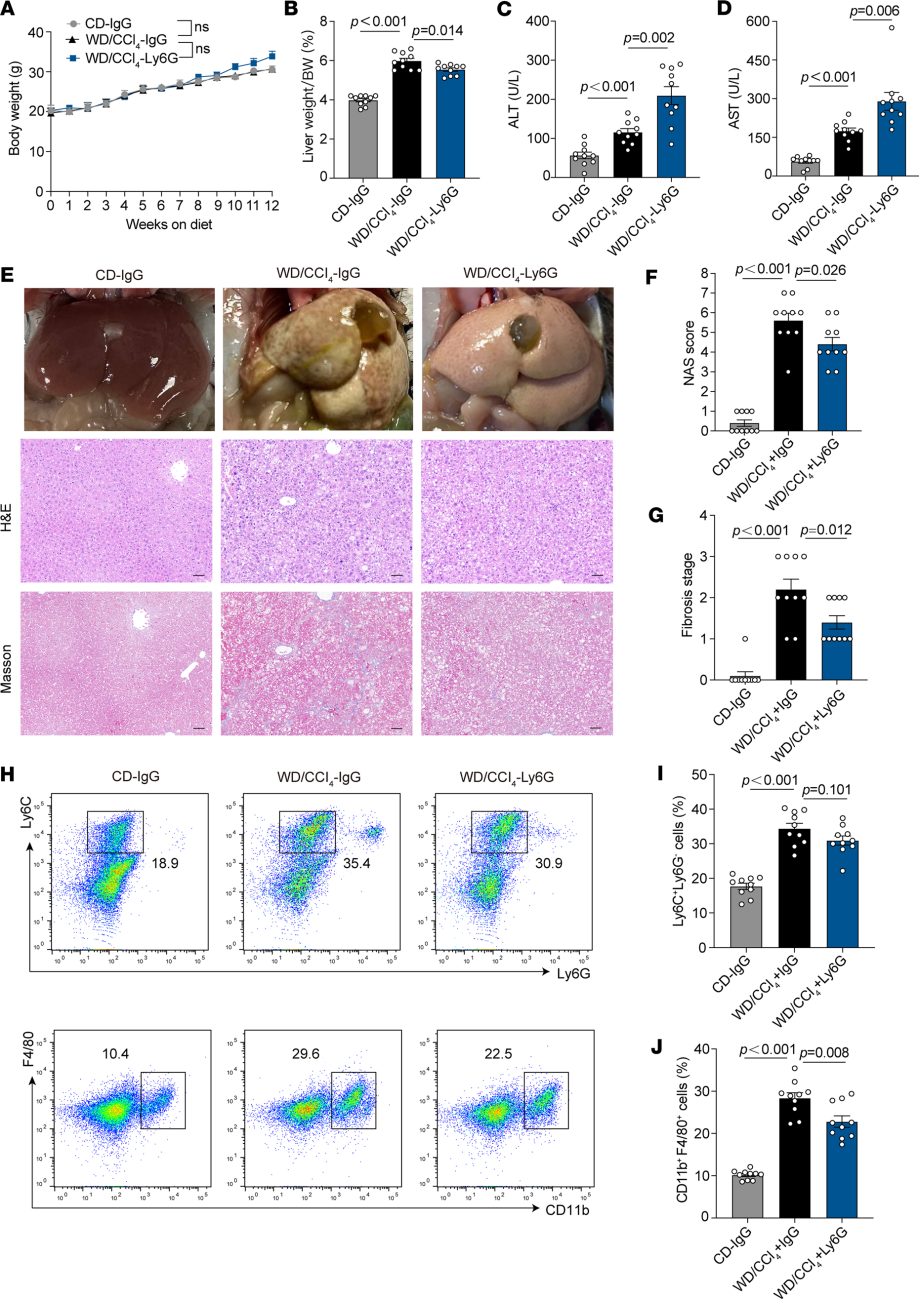
Effects of neutrophil depletion on liver under WD/CCl_4_ model. (**A**) Weekly monitored body weight of CD-IgG–, WD/CCl_4_–IgG–, and WD/CCl_4_–Ly6G–treated mice (10 mice per group). (**B**) Liver weight/body weight ratio of mice in each group. (**C**) Serum ALT levels across different groups. (**D**) Serum AST levels across different groups. (**E**) Representative liver sections showing phenotypic changes, lipid accumulation, and fibrosis, assessed by Masson staining. Scale bars: 50 μm. (**F**) NAS scores of each group. (**G**) Fibrosis stage of each group. (**H**–**J**) Flow cytometry analysis of CD45^+^ NPCs extracted from WD/CCl_4_–fed mouse livers, showing monocyte (Ly6C^+^Ly6G^–^) and macrophage (CD11b^+^F4/80^+^) populations, based on 10 mice per group. Statistical analyses were performed using 1-way ANOVA. Data are shown as the mean ± SEM.

In the WD/CCl_4_ model, body weight gain remained comparable between the control diet (CD) and WD/CCl_4_ groups, with neutrophil depletion having no significant effect on this parameter (Figure 3A). During the MASH stage, liver weight significantly increased; however, neutrophil depletion led to a slight reduction in liver weight (Figure 3B). Importantly, levels of ALT and AST were significantly elevated following neutrophil depletion, indicating exacerbated hepatocyte damage (Figure 3, C and D). Pathological analysis further revealed a reduction in MASH severity, as evidenced by decreased NAS scores and lower fibrosis grades (Figure 3, E–G). Flow cytometric analysis demonstrated a reduction in liver infiltration by monocytes and macrophages following neutrophil depletion (Figure 3, H–J). Taken together, these findings suggest that neutrophil depletion alleviates liver steatosis and fibrosis, while potentially exacerbating hepatocyte injury in the context of MASH.

### Single-cell RNA sequencing identifies 5 distinct neutrophil subsets infiltrating the human liver, with PAD4^+^ neutrophils specifically associated with the development of MASLD.

To investigate alterations in immune cell composition during the progression of MASLD, we performed single-cell RNA sequencing (scRNA-Seq) on non-parenchymal cells isolated from patients with MASL (*n* = 6) and MASH (*n* = 6). After filtering out dead cells and doublets, we obtained a total of 121,020 cell transcriptomes (51.1% from MASL and 48.9% from MASH; Supplemental Figure 2A). Cell populations were annotated based on canonical marker genes (Supplemental Figure 2B). The analysis revealed 9 major cell types: B cells, cholangiocytes, hepatocytes, mast cells, myeloid cells, plasma cells, stromal cells, T and NK cells, and plasmacytoid dendritic cells (pDCs) (Supplemental Figure 2C). Notably, the frequency of myeloid cells was significantly elevated in MASH compared with MASL, while the frequencies of other cell types remained relatively unchanged (Supplemental Figure 2D).

**Figure 4:**
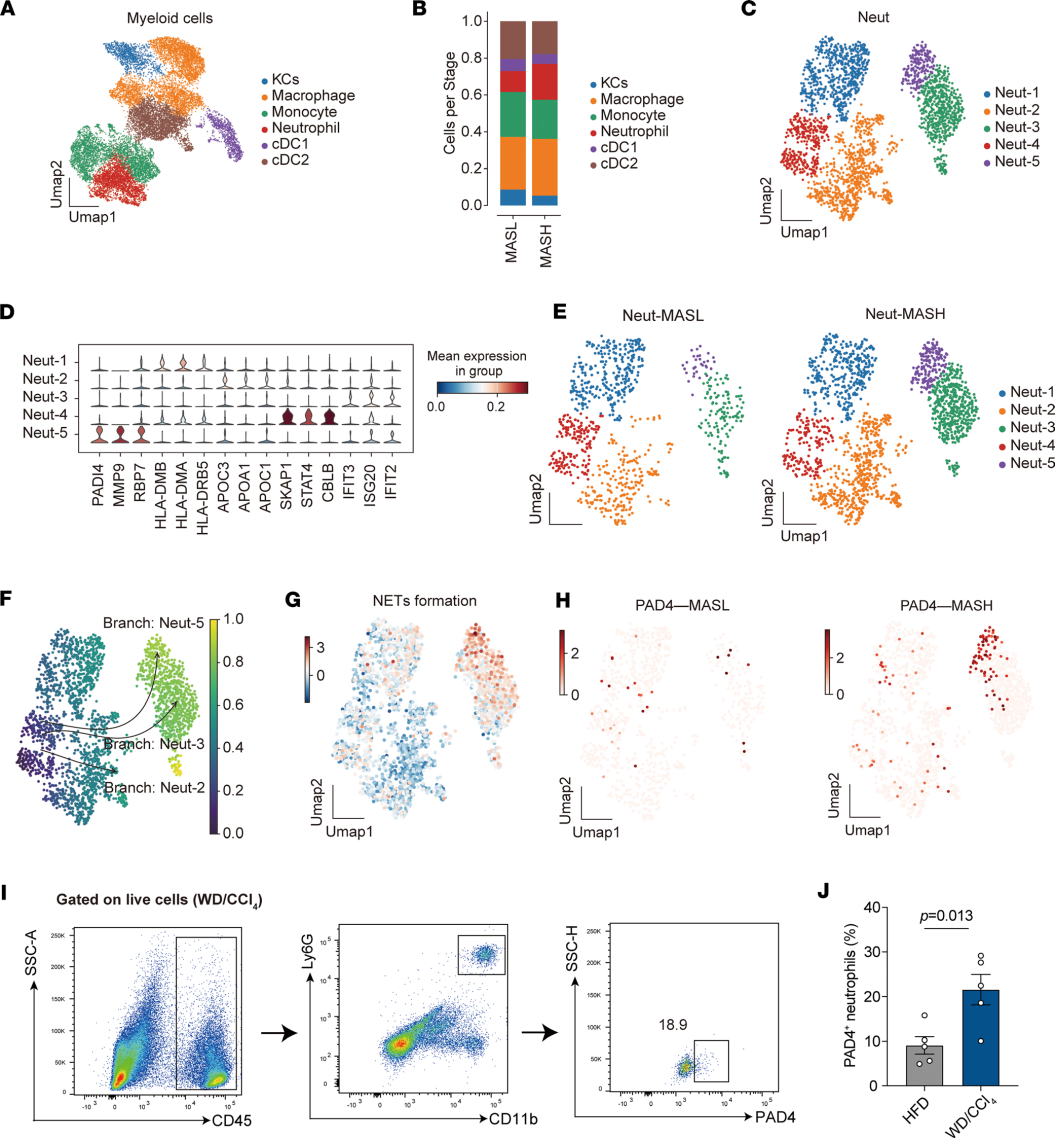
scRNA-Seq reveals neutrophil heterogeneity in human MASLD liver, with PAD4^+^ neutrophils correlating with MASH development. (**A**) Uniform manifold approximation and projection (UMAP) analysis of transcriptional profiles of myeloid cells (*n* = 15,479) from human MASL (*n* = 6) and MASH (*n* = 6) livers, with each cluster represented by a different color. (**B**) Bar plots showing the proportion of 6 myeloid cell subpopulations in human MASL and MASH livers. (**C**) UMAP analysis of transcriptional profiles of neutrophils (*n* = 2,428) from human MASL (*n* = 6) and MASH (*n* = 6) livers, with each cluster represented by a different color. (**D**) Violin plots showing expression levels and distribution of representative marker genes across neutrophil clusters. (**E**) UMAP analysis showing the distribution of 5 neutrophil subclusters in human MASL and MASH livers. (**F**) Pseudotime trajectory analysis of neutrophils using Palantir software (https://palantir.readthedocs.io), with cells colored by pseudotime progression. (**G**) UMAP showing NET formation signature scores in neutrophils, with color intensity reflecting scaled signature scores. Seurat (v4.3) AddModuleScore function was used to calculate NET gene signature scores. (**H**) UMAP of neutrophils colored by PAD4 cluster in MASL and MASH livers. (**I**) Flow cytometry analysis of PAD4^+^ neutrophils from livers of WD/CCl_4_–fed mice. (**J**) The proportion of PAD4^+^ neutrophils within the liver-infiltrating neutrophils in HFD- and WD/CCl_4_–fed mice. Statistical analyses were performed using 2-tailed Student’s *t* test. Data are shown as the mean ± SEM.

We next examined neutrophils in MASL- and MASH-affected livers. Myeloid cells were classified into 6 subclusters, including Kupffer cells (KCs), macrophages, monocytes, neutrophils, and 2 dendritic cell clusters (Figure 4A and Supplemental Figure 3A). The proportion of neutrophils and macrophages significantly increased in MASH, with neutrophil expansion being more pronounced (19.6% vs. 11.4%, *P* = 0.047) (Figure 4B). Differential gene expression analysis revealed upregulation of interferon-related and proinflammatory genes, such as IFITM3, HSPA1A, and CD69, in MASL (Supplemental Figure 3, B and C). In contrast, genes associated with neutrophil activation and phagocytosis, including S100A8, S100A9, and SOD2, were upregulated in MASH (Supplemental Figure 3, B and D). These findings indicate increased and activated neutrophil infiltration in MASH.

Further analysis of the 2,428 neutrophils across all samples (34.7% from MASL, 65.3% from MASH) using uniform manifold approximation and projection (UMAP) identified 5 distinct neutrophil clusters, with each cluster containing cells from all donors, indicating that there is no donor-specific transcriptomic profile (Figure 4C). Neutrophil populations were annotated based on the expression levels of marker genes (Figure 4D). The MASH group showed significantly higher proportions of Neut-3 and Neut-5 compared with MASL (Figure 4E and Supplemental Figure 4A). Notably, PAD4, MMP-9, and RBP7 were highly expressed in Neut-5, distinguishing it from the other clusters (Figure 4D). Kyoto Encyclopedia of Genes and Genomes (KEGG) analysis revealed that genes in Neut-5 were enriched in pathways related to NET formation and IL-17 signaling (Supplemental Figure 4B). Gene Ontology (GO) analysis further indicated enrichment for stress responses and cell chemotaxis in Neut-5 (Supplemental Figure 4C). Pseudotemporal trajectory analysis suggested differentiation from Neut-4 into Neut-2, Neut-3, or Neut-5 (Figure 4F).

After further validation, gene signature scoring confirmed that NET formation was predominantly localized within the Neut-5 subset (Figure 4G). PAD4, a marker gene for Neut-5, exhibited significantly increased expression in the MASH group (Figure 4H). Flow cytometry analysis corroborated these findings, revealing a significantly higher frequency of PAD4^+^ neutrophils in mice affected by MASH compared with those with MASL (Figure 4, I and J, and Supplemental Figure 4D). Collectively, our results suggest that human liver-infiltrating neutrophils constitute a heterogeneous population composed of 5 distinct subsets, with PAD4^+^ neutrophils potentially playing a role in the progression of MASLD through NET formation.

### Inflammatory cytokines in MASH-affected livers drive the increased expression of PAD4^+^ neutrophils and the formation of NETs.

**Figure 5:**
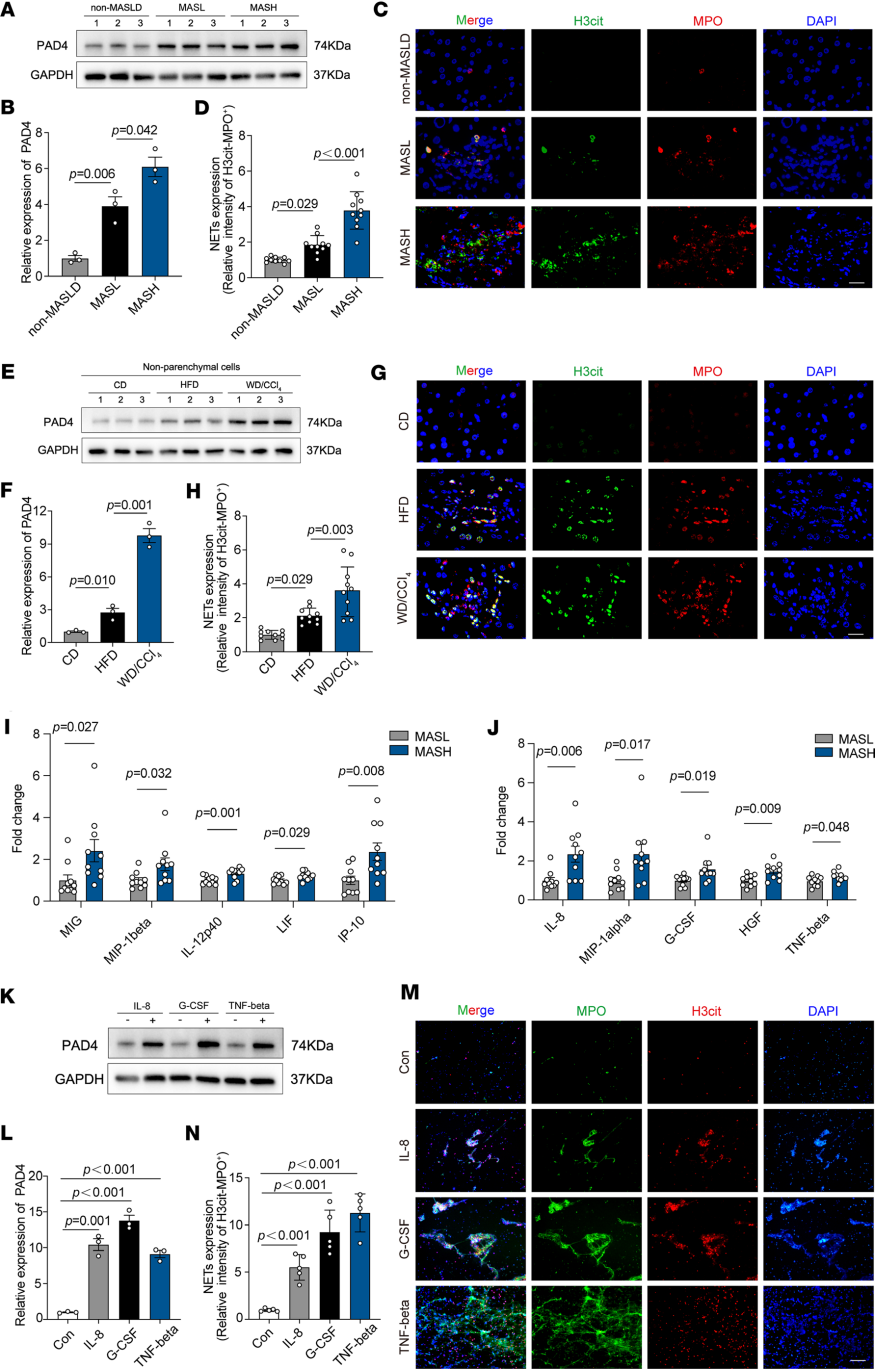
Inflammatory cytokines in MASH livers promote increased expression of PAD4^+^ neutrophil and NET formation. (**A**) Western blot analysis of PAD4 expression in liver tissues from non-MASLD, MASL, and MASH patients (*n* = 3 per group). (**B**) Quantitative grayscale analysis of PAD4 protein bands from Western blot. (**C**) Representative immunofluorescence images of citrullinated histone H3 (H3Cit) and MPO staining in liver tissues from non-MASLD, MASL, and MASH patients. NETs are identified by costaining with H3Cit, MPO, and DAPI, with red fluorescence indicating neutrophils. Scale bar: 20 μm. (**D**) Quantification of H3Cit^+^MPO^+^ staining intensity using ImageJ software. (**E**) Western blot analysis of PAD4 expression in non-parenchymal liver cells from CD-, HFD-, and WD/CCl_4_–fed mice (*n* = 3 per group). (**F**) Quantitative grayscale analysis of PAD4 protein bands in non-parenchymal liver cells. (**G**) Representative immunofluorescence images of H3Cit and MPO staining in livers from CD-, HFD-, and WD/CCl_4_–fed mice. NETs are identified by costaining with H3Cit, MPO, and DAPI, with red fluorescence indicating neutrophils. Scale bar: 20 μm. (**H**) Quantification of H3Cit^+^MPO^+^ staining intensity in mouse liver tissues using ImageJ software. (**I** and **J**) Luminex assay (Bio-Rad Laboratories) quantification of inflammation-related cytokines in liver tissues from MASL (*n* = 10) and MASH (*n* = 10) patients. Statistically significant cytokines are highlighted. (**K**) Western blot analysis of PAD4 expression in neutrophils stimulated with IL-8, G-CSF, and TNF-β. (**L**) Quantitative grayscale analysis of PAD4 protein bands in stimulated neutrophils. (**M**) Immunofluorescence assessment of NET formation potential in neutrophils stimulated with IL-8, G-CSF, and TNF-β (H3Cit in red, MPO in green, DAPI in blue). Scale bar: 200 μm. (**N**) Quantification of H3Cit^+^MPO^+^ staining intensity after IL-8, G-CSF, and TNF-β stimulation using ImageJ software. Statistical analyses were performed using 1-way ANOVA. Data are shown as the mean ± SEM.

To validate the role of PAD4^+^ neutrophils in the progression of MASLD, we assessed PAD4 expression in liver biopsies from patients at various stages of MASLD. PAD4 expression was significantly elevated in the MASH stage (Figure 5, A and B). PAD4-mediated histone citrullination is widely recognized as a crucial step in NET formation (14). To further investigate, we performed immunofluorescence staining for myeloperoxidase (MPO) and citrullinated histone H3, specific markers for neutrophils and NETosis. NET formation was minimally detected in non-MASLD and MASL tissues, but was prominently present in MASH tissues (Figure 5, C and D). These findings were further corroborated in mouse models, where WD/CCl_4_ mice exhibited significantly higher levels of hepatic NETs and PAD4 compared with CD- or HFD-fed mice (Figure 5, E–H). Together, these results suggest that PAD4^+^ neutrophils and NET formation are closely associated with the progression of MASLD.

Neutrophils function by responding to inflammatory signals and migrating to sites of injury or infection (15). To investigate the causes of elevated PAD4^+^ neutrophil and NET formation in MASH livers, we examined the expression of inflammatory cytokines in human liver samples. Analysis of 48 cytokines in MASL and MASH patient livers revealed 10 cytokines that were significantly elevated in MASH: MIG, MIP-1β, IL-12p40, LIF, IP-10, IL-8, MIP-1α, G-CSF, HGF, and TNF-β (Figure 5, I and J, and Supplemental Table 3). Among these, IL-8, G-CSF, and TNF-β have been identified as inducers of NET formation (16–18). Fluorescence analysis confirmed that IL-8, G-CSF, and TNF-β promoted increased PAD4 expression and NET formation in vitro (Figure 5, K–N). No significant differences were observed in most other inflammatory cytokines (Supplemental Figure 5, A–E). Collectively, these findings demonstrate that inflammatory cytokines in MASH livers contribute to the elevated expression of PAD4^+^ neutrophils and enhanced NET formation, underscoring the role of the inflammatory microenvironment in disease progression.

### Inhibition of PAD4^+^ neutrophils slows fibrosis progression in MASH livers without exacerbating liver damage.

**Figure 6:**
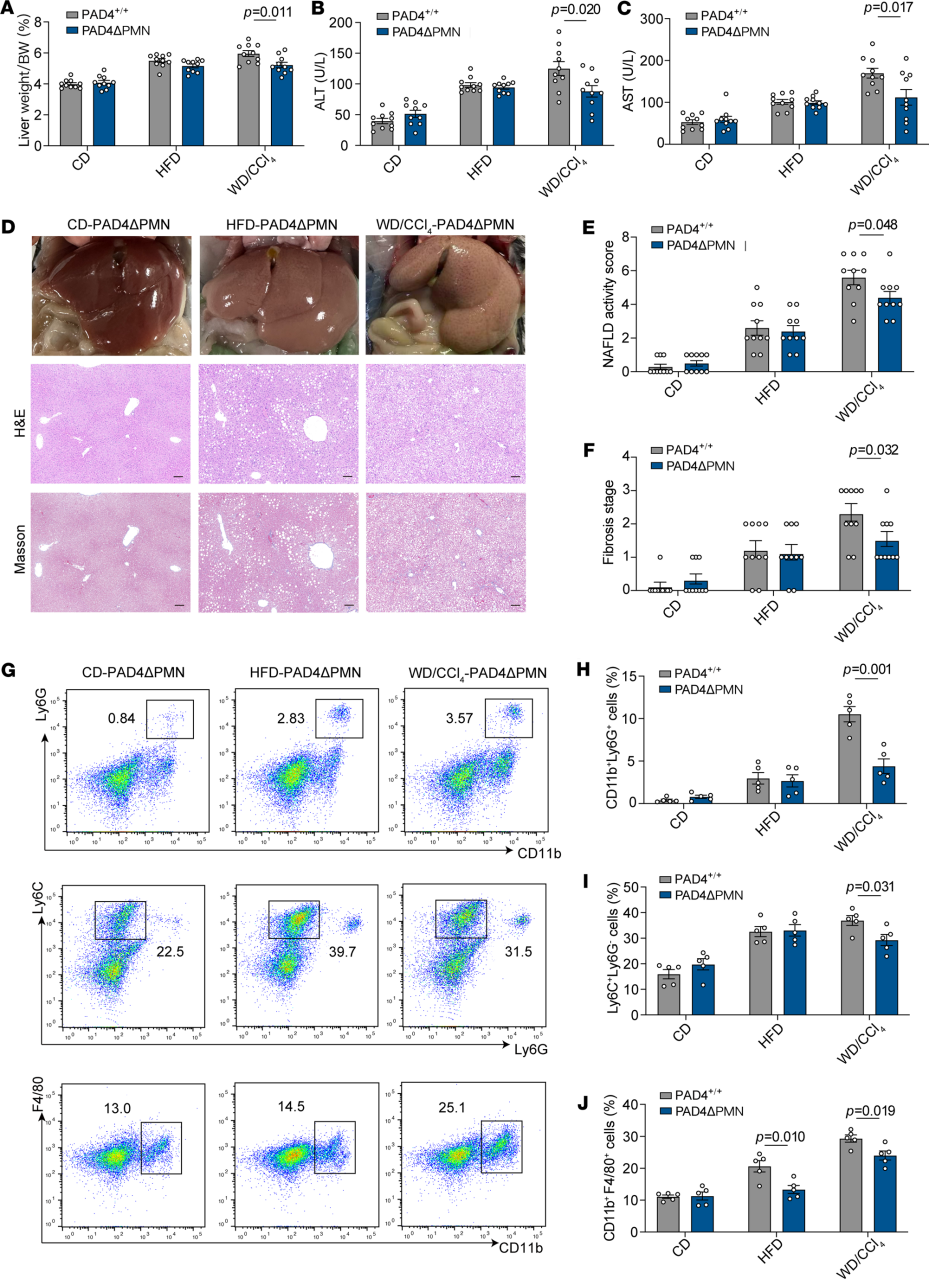
Effects of inhibiting PAD4^+^ neutrophil and NET formation on liver under CD, HFD, and WD/CCl_4_ models. (**A**) Liver weight/body weight ratio of mice in each group. (**B**) Serum ALT levels across different groups. (**C**) Serum AST levels across different groups. (**D**) Representative liver sections showing phenotypic changes, lipid accumulation, and fibrosis, assessed by Masson staining. Scale bars: 100 μm. (**E**) NAS scores of each group. (**F**) Fibrosis stage of each group. (**G**–**J**) Flow cytometry analysis of CD45^+^ NPCs extracted from the livers of PAD4ΔPMN mice and PAD4^+/+^ mice fed different models, showing neutrophil (CD11b^+^Ly6G^+^), monocyte (Ly6C^+^Ly6G^–^), and macrophage (CD11b^+^F4/80^+^) populations. Data are presented as mean ± SEM, based on 10 independent experiments. Statistical analyses were performed using 1-way ANOVA. Data are shown as the mean ± SEM.

To investigate the role of PAD4^+^ neutrophils in MASH, we developed mice with neutrophil-specific knockout of PAD4 (PAD4^fl/fl^; MRP8-Cre, PAD4ΔPMN) (Supplemental Figure 6A). This model has been well validated in multiple published studies to achieve efficient and specific deletion of PAD4 in neutrophils (19–22). In the HFD and WD/CCl_4_ models, weight changes in PAD4ΔPMN mice were comparable to those observed in CD models (Supplemental Figure 6B). The neutrophil-specific deficiency of PAD4 in the offspring was confirmed by quantitative reverse transcription PCR (qRT-PCR), verifying successful gene deletion (Supplemental Figure 6C). PAD4ΔPMN mice exhibited normal peripheral neutrophil counts (Supplemental Figure 6D). In the WD/CCl_4_–induced MASH model established in PAD4ΔPMN mice, NET formation in the liver was almost completely abolished (Supplemental Figure 6E). Neutrophils from PAD4ΔPMN mice exhibited minimal NET formation. In the HFD model, no significant differences in liver weight were noted between PAD4ΔPMN and PAD4^+/+^ mice. However, in the WD/CCl_4_ model, liver weight was significantly reduced in PAD4ΔPMN mice compared with PAD4^+/+^ mice (Figure 6A). Furthermore, serum ALT and AST levels were significantly lower in PAD4ΔPMN mice under the WD/CCl_4_ model, suggesting reduced liver injury, while no significant differences were observed in the HFD model (Figure 6, B and C). These results indicate that the inhibition of PAD4^+^ neutrophils in the liver does not exacerbate liver damage and may reduce hepatic inflammation in the context of MASH.

Histopathological analysis revealed reduced severity of MASH in PAD4ΔPMN mice, as indicated by lower NAS scores and fibrosis grades compared with PAD4^+/+^ mice (Figure 6, D–F). Flow cytometric analysis showed a significant reduction in liver infiltration by neutrophils, monocytes, and macrophages in PAD4ΔPMN mice, suggesting that inhibition of PAD4^+^ neutrophil and NET formation limits inflammatory cell infiltration (Figure 6, G–J). To determine whether the observed effects were due to reduced NET formation or impaired neutrophil recruitment to the liver, we performed neutrophil adoptive cell transfer (ACT). Bone marrow cells were isolated from PAD4^+/+^ and PAD4ΔPMN mice, and bone marrow–derived neutrophils (BMDNs) were purified using anti-Ly6G. The results showed that liver fibrosis of PAD4ΔPMN mice was aggravated after injection of BMDNs from PAD4^+/+^ mice. However, the degree of liver fibrosis in PAD4^+/+^ mice did not change after injection of BMDNs from PAD4ΔPMN mice (Supplemental Figure 6, F and G). Collectively, these findings demonstrate that inhibiting PAD4^+^ neutrophil and NET formation slows fibrosis progression in MASH without exacerbating liver injury or inflammation.

### PAD4^+^ neutrophils promote HepSC activation through NET formation and the activation of the MAPK pathway.

**Figure 7:**
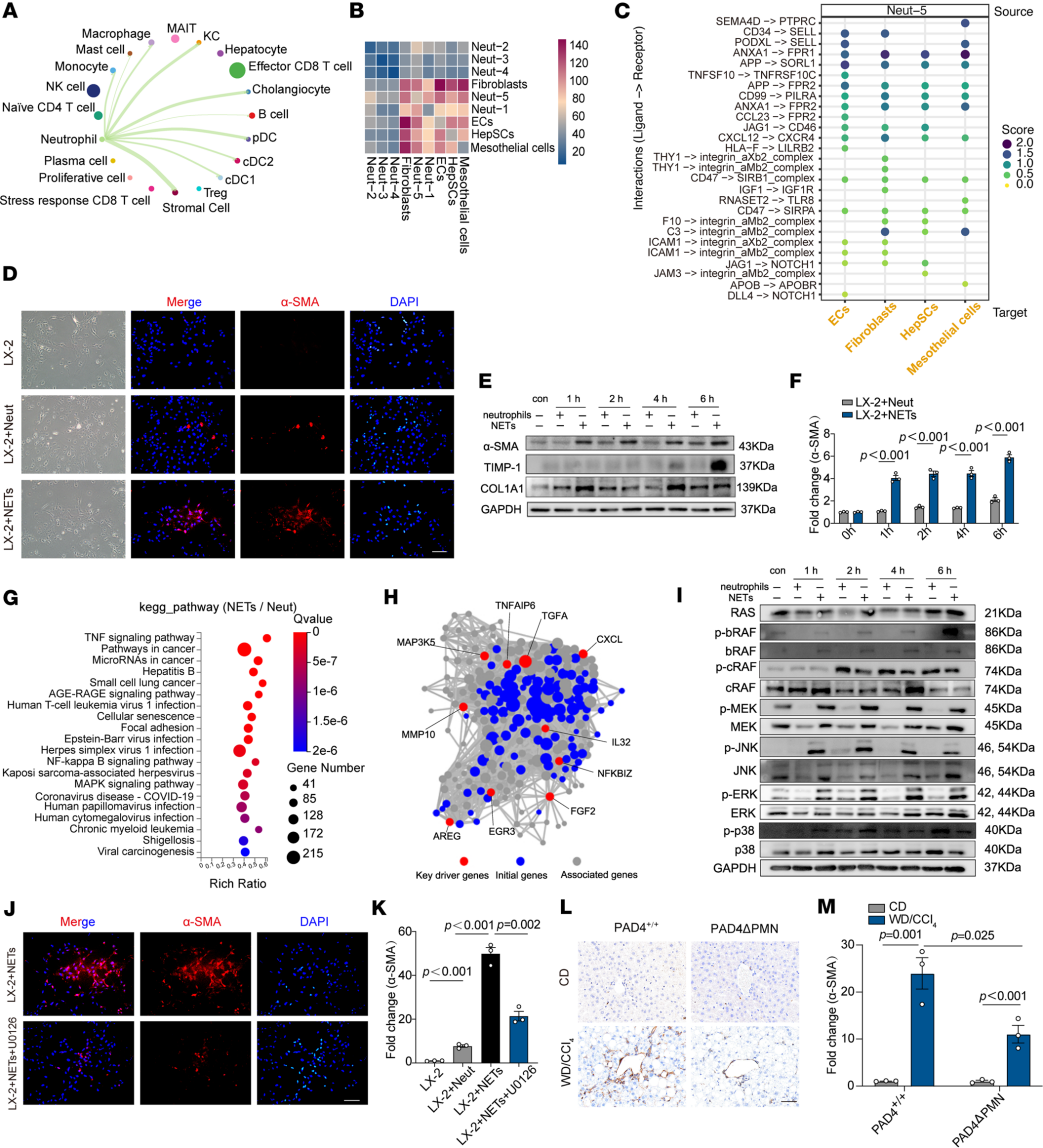
PAD4^+^ neutrophils promote HepSC activation via NET formation and MAPK pathway activation. (**A**) Networks revealing interactions among neutrophils and various cell types. The thicker the line, the higher the number of interactions among cell types. (**B**) Heatmap exhibiting cell-cell communication calculated by CellphoneDB (www.cellphonedb.org). Color represents cumulative count of significant ligand-receptor pairs across different cell types. (**C**) Dot plot showing significant ligand-receptor interactions (*P* < 0.05) between Neut-5 and stromal cell subtypes. The data are segmented with Neut-5 as source cells, according to CellphoneDB. Stromal cell subtypes are shown on the *x* axis, and ligand-receptor pairs on the *y* axis. Dot size and color intensity represent mean score. (**D**) Neutrophils pretreated with IL-8, G-CSF, and TNF-β and induced NETs were cocultured with LX-2 HepSCs for 6 hours. Immunofluorescence detected LX-2 cell activation (α-SMA in red, DAPI in blue). Scale bar: 100 μm. (**E**) Time course study (0, 1, 2, 4, 6 hours) of neutrophils or NETs cocultured with LX-2. LX-2 activation markers (α-SMA, TIMP-1, COL1A1) were analyzed using Western blot. (**F**) Quantitative grayscale analysis of α-SMA protein bands from Western blots. (**G**) Dot plot showing significant KEGG pathway terms. (**H**) Key driver gene analysis of differentially expressed genes was performed to identify genes with major regulatory roles. (**I**) Time course studies (0, 1, 2, 4, 6 hours) of neutrophils or NETs cocultured with LX-2. Key proteins in the MAPK pathway were analyzed by Western blotting. (**J**) Coculturing of NETs and LX-2 cells with the MAPK pathway inhibitor U0126 added; subsequent changes in LX-2 activation were assessed via immunofluorescence (α-SMA in red, DAPI in blue). Scale bars: 100 μm. (**K**) Quantification of α-SMA^+^ staining intensity using ImageJ software. (**L**) IHC detection of α-SMA in liver sections from PAD4^+/+^ and PAD4ΔPMN mice before and after WD/CCl_4_ dietary intervention. Scale bar: 50 μm. (**M**) Quantification of α-SMA^+^ staining intensity using ImageJ software. Statistical analyses were performed using 1-way ANOVA. Data are shown as the mean ± SEM.

To elucidate the role of liver-infiltrating PAD4^+^ neutrophils in MASH progression, we investigated intercellular communication involving neutrophils. Cell-cell communication analysis was conducted to assess the potential and strength of ligand-receptor interactions among various cell types. Notably, neutrophils exhibited the most prominent interactions with stromal cells (Figure 7A). Further analysis of the interactions between neutrophil subpopulations and stromal cells revealed that PAD4^+^ neutrophils (Neut-5) had significantly stronger connections with stromal cell types, including mesothelial cells, HepSCs, fibroblasts, and endothelial cells (ECs), compared with other neutrophil subpopulations (Figure 7, B and C).

Given the pivotal role of HepSCs in fibrosis formation, we investigated whether PAD4^+^ neutrophils could induce HepSC activation. Peripheral blood neutrophils from MASH patients were treated with IL-8, G-CSF, and TNF-β to promote the formation of PAD4^+^ neutrophils. Coculture experiments were then performed with PAD4^+^ neutrophils, NETs, and LX-2, a human HepSC line. NETs significantly enhanced LX-2 activation, while direct coculture with neutrophils alone exhibited minimal effects (Figure 7, D and K). Western blot analysis revealed upregulation of LX-2 activation markers, including α-SMA, TIMP-1, and COL1A1, in response to NETs compared with neutrophils alone (Figure 7, E and F, and Supplemental Figure 7, A and B).

To further investigate the molecular mechanisms underlying LX-2 activation, RNA sequencing was performed on LX-2 cells exposed to NETs. Differential expression analysis revealed upregulation of 539 genes and downregulation of 165 genes (Supplemental Figure 7C). GO enrichment analysis indicated that the differentially expressed genes were predominantly involved in inflammatory responses, cytokine-mediated signaling, and the regulation of cell proliferation (Supplemental Figure 7D). KEGG pathway analysis revealed significant enrichment in pathways such as TNF signaling, cytokine-receptor interactions, and NF-κB signaling, with notable involvement of the MAPK pathway (Figure 7G), which is known to regulate cell growth, differentiation, and responses to environmental stress (23).

Among the upregulated genes, key drivers such as CXCL6, FGF-2, and MMP-10 were identified as being associated with the activation of the MAPK pathway (Figure 7H). Western blot analysis confirmed MAPK pathway activation following NET treatment in LX-2 cells (Figure 7I and Supplemental Figure 7, E–G). To investigate the role of MAPK signaling in LX-2 activation, we used the MAPK inhibitor U0126. Inhibition of the MAPK pathway resulted in a significant reduction in LX-2 activation (Figure 7, J and K). Following WD/CCl_4_ dietary intervention, PAD4ΔPMN mice exhibited significantly reduced expression of α-SMA compared with PAD4^+/+^ mice (Figure 7, L and M). Collectively, these findings suggest that PAD4^+^ neutrophils promote HepSC activation through NET formation and subsequent MAPK pathway activation.

### NET-DNA activates the MAPK pathway in a TAOK1-dependent manner, thereby promoting liver fibrosis.

**Figure 8:**
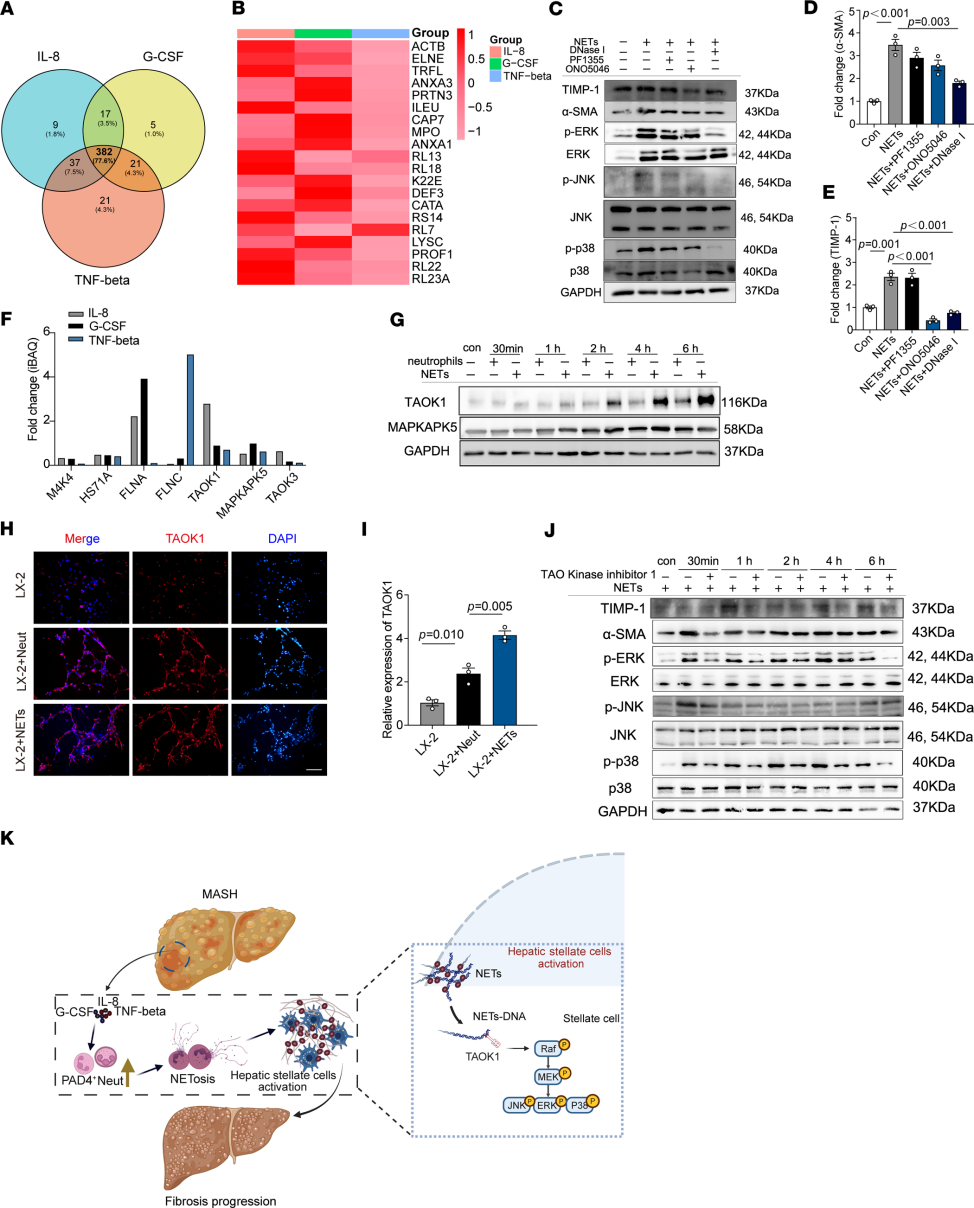
The critical role of NET-DNA and TAOK1 interaction in MAPK pathway activation. (**A**) Neutrophils isolated from MASH patient peripheral blood were induced to form NETs upon stimulation with IL-8, G-CSF, and TNF-β. Proteomic analysis using liquid chromatography/mass spectrometry (LC-MS) identified the amounts of proteins in these NETs, which are depicted in a Venn diagram. (**B**) A heatmap illustrates the top 20 protein components identified in the NETs by LC-MS, highlighting key proteins associated with NET formation. (**C**) Western blot analysis was performed to assess the impact of MPO inhibitor (PF1355), NE inhibitor (ONO5046), and DNase I treatment on MAPK pathway activation in LX-2 cells exposed to NETs. (**D** and **E**) Quantitative grayscale analysis of Western blot bands for α-SMA and TIMP-1 expression in response to NETs and inhibitors. (**F**) LC-MS analysis of enriched proteins associated with the MAPK pathway following NET-DNA pull-down, with key MAPK pathway components highlighted. (**G**) Coculture of neutrophils or NETs with LX-2 cells for varying durations (0, 30 minutes, 1, 2, 4, and 6 hours), followed by Western blot detection of TAOK1 and MAPKAPK5 expression levels. (**H**) Immunofluorescence microscopy showed changes in TAOK1 expression during co-cultivation of NETs and LX-2 cells (TAOK1 shown in red, DAPI for cell nuclei in blue). Scale bar: 100 μm. (**I**) Quantification of TAOK1^+^ staining intensity using ImageJ software, showing the increase in TAOK1 expression in LX-2 cells following NET exposure. (**J**) Western blot analysis after TAOK1 inhibitor treatment to examine the impact of NETs on MAPK pathway activation in LX-2 cells. (**K**) Schematic diagram illustrating the mechanism by which NET-DNA activates the MAPK pathway through TAOK1 interaction, promoting HepSC activation and fibrosis (created with BioRender; biorender.com). Statistical analyses were performed using 1-way ANOVA. Data are shown as the mean ± SEM.

To investigate the role of NETs in activating the MAPK pathway, we first analyzed their protein composition. Neutrophils were isolated from peripheral blood samples of MASH patients and induced to form NETs using IL-8, G-CSF, and TNF-β. Proteomic analysis of NETs by liquid chromatography–mass spectrometry (LC-MS) identified a total of 492 proteins, with coexpressed proteins accounting for the majority (77.6%) (Figure 8A). Among these, we identified several known NET protein components, such as PRTN3, ELANE, and MPO (Figure 8B), which is consistent with previous reports indicating that NETs primarily function through granular proteins like ELANE and MPO (24).

Western blot analysis demonstrated that NET stimulation led to the phosphorylation of ERK, JNK, and p38, along with an increase in the expression of α-SMA and TIMP-1. Interestingly, inhibition of ELANE and MPO using specific inhibitors (ONO5046 and PF1355) did not suppress the activation of the MAPK pathway or reduce the expression of fibrosis-related proteins. However, treatment with DNase I inhibited the MAPK pathway and significantly decreased the expression of fibrosis-related proteins (Figure 8, C–E, and Supplemental Figure 8A). These findings suggest that NETs function through NET-DNA, which activates HepSCs via the MAPK pathway.

To further explore the underlying mechanism, we extracted membrane proteins from LX-2 cells and coincubated them with NET-DNA. Interacting proteins were identified through DNA pull-down assays followed by mass spectrometry (Supplemental Figure 8B). A total of 414 interacting proteins were detected, including several associated with the MAPK pathway (Supplemental Figure 8, C and D). Among these, TAOK1 and MAPKAPK5 exhibited higher and more stable expression levels (Figure 8F). Western blot analysis confirmed that NET exposure increased TAOK1 expression in LX-2 cells (Figure 8G and Supplemental Figure 8E). In vitro immunofluorescence further supported these findings, showing enhanced TAOK1 expression in LX-2 cells following NET exposure (Figure 8, H and I).

To verify the role of TAOK1 in MAPK pathway activation, we treated LX-2 cells with a specific TAOK1 inhibitor (TAO kinase inhibitor 1). This treatment resulted in the inhibition of the MAPK pathway and a reduction in the expression of fibrosis-related proteins (Figure 8J and Supplemental Figure 8F). Collectively, these data indicate that NET-DNA activates the MAPK pathway through TAOK1 binding, thereby promoting HepSC activation and liver fibrosis.

## Discussion

MASLD is a multifactorial disorder, with MASH serving as a crucial stage in the progression of liver inflammation and fibrosis. Immune cells play a pivotal role in the progression from MASLD to MASH and, ultimately, to cirrhosis (5). This study provides new insights into the role of neutrophils in MASLD pathogenesis, specifically in the transition from MASH to liver fibrosis (Figure 8K). Recent studies have suggested that neutrophils exacerbate hepatic inflammation and fibrosis through mechanisms such as NET formation and the release of proteolytic enzymes (6, 25, 26). Our findings underscore the heterogeneity of neutrophils in MASH and provide mechanistic insights into their role in NET formation and HepSC activation, particularly the interactions between liver fibrosis and PAD4^+^ neutrophils. This highlights PAD4^+^ neutrophils as a potential therapeutic target for MASH treatment.

Neutrophils are among the first immune cells to be recruited to the liver in response to cellular stress and damage caused by lipid accumulation. Once they infiltrate the liver, neutrophils contribute to local inflammation through the release of various proinflammatory cytokines, chemokines, and reactive oxygen species (27–29). Consequently, neutrophil infiltration serves as a key histopathological feature of MASH. While the pathological and clinical relevance of neutrophils remains largely unknown, studies have focused on the neutrophil-to-lymphocyte ratio, which integrates information about the inflammatory environment and physiological stress (30, 31). Our analysis of MASLD liver tissues in both humans and mice revealed that neutrophil counts were positively correlated with MASLD severity, particularly fibrosis. Additionally, the correlation between neutrophil infiltration and clinical parameters such as BMI and liver enzymes complements existing knowledge about immune cell involvement in MASH. These findings extend current understanding by emphasizing the complex and significant role neutrophils play in MASH progression, thereby enhancing our comprehension of the interactions between immune responses and liver pathology in MASLD.

Neutrophils, as a critical component of the immune system, play a pivotal role in the immune microenvironment of MASH (32–34). Therefore, regulating neutrophil function or reducing their accumulation in the liver could offer a promising strategy for treating MASH. In recent years, several animal models and in vitro studies have explored ways to reduce inflammatory responses in MASH by targeting neutrophil chemokines or their receptors (6, 33–36). Notably, targeting neutrophil chemokines such as CCL2 and CCL5 or their receptors CCR2 and CCR5 to reduce inflammation in MASH has garnered significant research attention. However, while the CCR2/5 antagonist cenicriviroc shows broad antiinflammatory effects, phase III clinical trials revealed no significant difference in liver fibrosis improvement between the treatment and placebo groups (37). Recent studies have indicated that targeting neutrophils with CXCR2 small-molecule inhibitors can enhance the effectiveness of immune checkpoint inhibitors in treating MASH-related hepatocellular carcinoma (38). In our study, we used the Ly6G antibody to deplete neutrophils in a mouse model and observed that neutrophil depletion inhibited the progression of hepatic steatosis and fibrosis, but also exacerbated liver damage in MASH. This aligns with studies demonstrating that neutrophil depletion in drug-induced acute liver injury models impedes macrophage turnover during liver repair, resulting in elevated serum ALT levels and increased liver necrosis (39). These findings suggest that the long-term effects and safety of neutrophil activity inhibition or neutrophil recruitment to the liver require further investigation.

Growing evidence has identified the existence of distinct neutrophil subtypes in circulation or within tissues under both physiological and pathological conditions (40–42). Discrete microenvironments can modify neutrophil function and behavior, contributing to their heterogeneity. Factors such as rapid neutrophil aging, their short lifespan, and mechanical responses during entry and exit from capillaries further contribute to this heterogeneity (43). In human and mouse liver tissues, the role of different neutrophil subsets in disease is complex, as some subsets contribute to disease progression while others may inhibit it. Cho et al. observed a unique low-density neutrophil population in individuals with alcohol-associated hepatitis and alcohol-fed mice, which was absent in healthy controls (12). In acute liver failure models, the downregulation of CXCR2^+^ neutrophils upon activation suggests their involvement in infiltration (44). Additionally, studies have shown that the number of IL-17^+^ neutrophils in fibrotic septa and portal areas strongly correlates with the stages of fibrosis in chronic viral hepatitis (45). However, neutrophils are less abundant in MASH liver tissue and are often underrepresented in single-cell datasets (46). Our study provides a preliminary analysis of neutrophil roles in MASLD progression through scRNA-Seq, including subgroup and functional analyses. We found that human liver-infiltrating neutrophils form heterogeneous populations consisting of 5 distinct subsets, with PAD4^+^ neutrophils playing a critical role in MASH development, particularly through NET formation. The inflammatory environment in MASH livers, characterized by IL-8, G-CSF, and TNF-β, contributes to the accumulation of PAD4^+^ neutrophils. Inhibiting PAD4^+^ neutrophil and NET formation in mice with neutrophil-specific PAD4 knockout slows fibrosis progression in MASH without exacerbating liver injury or inflammation. Therefore, inhibiting PAD4^+^ neutrophils could emerge as an effective therapeutic approach for neutrophil-targeted therapy in liver fibrosis.

NETs, which form a web-like structure composed of DNA strands, histones, and granular proteins, have been increasingly recognized for their role in promoting non-infectious inflammation (47–49). Recent studies have detected NETs in MASH mouse models and human MASH livers, where they were associated with higher NAS scores and increased inflammation levels (50, 51). These studies in particular have found a strong link between NETs and MASH liver fibrosis (25, 52). For example, alcohol binges were shown to specifically increase NET formation in MASH livers in both mice and humans. NETs activate HepSCs through NLRP3 sensing in alcohol-induced MASH fibrosis, accelerating its progression (25). Another study revealed that NETs promote HepSC activation, proliferation, and migration by enhancing mitochondrial function and aerobic glycolysis to meet the bioenergetic demands of fibrosis (52). However, our study significantly extends these findings by elucidating the precise upstream molecular mechanism by which NETs — specifically their DNA component — activate HepSCs. Our observations confirm the role of NETs in HepSC activation, establishing a critical link in the cascade leading to liver fibrosis. Moreover, previous studies have not elucidated the effects of NET components on HepSC activation. Our findings reveal that NET-DNA plays a pivotal role in directly interacting with membrane proteins, thereby triggering HepSC activation. Additionally, NET-DNA activation of the MAPK pathway, particularly through TAOK1 interaction, unveils a mechanism of HepSC activation, further enhancing our understanding of the intracellular signaling pathways involved in liver fibrosis.

However, there are limitations to this study. First, the cross-sectional design of our human studies limits our ability to establish causality between neutrophil infiltration and MASLD progression. Longitudinal studies are required to better understand the temporal dynamics of neutrophil involvement in MASLD. Additionally, although we integrated single-cell sequencing results from our data with existing literature, the small sample size and low gene expression levels of neutrophils posed challenges for accurate subpopulation and functional analysis. Given the broad interactions predicted between neutrophils and other immune cell components, further functional studies are needed to identify the biomolecules mediating these communications and to clarify the mechanisms by which neutrophils promote liver fibrosis. Finally, because PAD4ΔPMN mice were generated with the MRP8-Cre line, potential transgene effects cannot be excluded. We also observed lower serum PAI-1 in PAD4ΔPMN mice (Supplemental Figure 8G); this is reported as an observation, and future confirmation with alternative Cre drivers or bone marrow chimeras will be needed to exclude transgene-related confounding.

In conclusion, our findings offer insights into the heterogeneity of neutrophils in MASH livers and highlight the critical role of PAD4^+^ neutrophils in the progression of MASLD. These PAD4^+^ neutrophils facilitate liver fibrosis progression through the formation of NETs. Targeting PAD4^+^ neutrophils or inhibiting NET formation may provide innovative therapeutic strategies for delaying or even reversing the progression of MASH-related liver fibrosis.

## Methods

Further information can be found in Supplemental Methods.

### Sex as a biological variable.

In the human cohort, sex was incorporated as a biological variable in both the study design and statistical analyses, with sex-based differences evaluated where appropriate. In the experimental arm, male C57BL/6J mice were used to investigate diet-induced MASLD. It should be noted that this study was restricted to male mice; therefore, the applicability of these findings to female mice remains to be determined.

### Human blood and liver tissue samples.

We enrolled 265 obese patients, comprising 63 non-MASLD and 202 MASLD individuals, undergoing bariatric metabolic surgery at the First Affiliated Hospital of Nanjing Medical University between January and December 2021. All participants gave written informed consent. Blood samples were collected before surgery, and liver biopsies were conducted during the operation. Exclusion criteria included obesity with viral hepatitis, serious comorbidities, heavy alcohol consumption, and drug abuse. This study adhered to the Helsinki Declaration guidelines and received approval from the Ethics Committee of the First Affiliated Hospital of Nanjing Medical University (2017-SR-171-A2).

### Liver histology.

Two experienced pathologists independently scored liver biopsies using the blind method based on the NASH Clinical Research Network (NASH CRN) scoring system (53). The NAFLD activity score (NAS) comprises steatosis (0 to 3), lobular inflammation (0 to 3), and ballooning (0 to 2). MASLD is characterized by steatosis in at least 5% of hepatocytes. The liver biopsy cohort was categorized for analysis into 3 groups: (a) non-MASLD: steatosis <5%; (b) non-MASH: steatosis ≥5% and NAS ≤4; (c) MASH: steatosis ≥5% and NAS ≥5. Fibrosis scores were assigned as follows: 0, none; 1, perisinusoidal or periportal fibrosis; 2, both perisinusoidal and portal/periportal fibrosis; 3, bridging fibrosis; and 4, cirrhosis.

Neutrophils were identified histopathologically using hematoxylin and eosin staining, complemented by immunohistochemical (IHC) techniques. For IHC, an anti-myeloperoxidase (anti-MPO) antibody was used. Staining was performed on formalin-fixed, paraffin-embedded tissues using established protocols. Neutrophils were identified by their segmented nuclei and granule-rich cytoplasm with a reddish hue. Six high-power fields at ×400 magnification and 0.55 μm field diameter were sequentially examined by 2 experienced histologists.

### Animals and animal models.

Mice were housed at Nanjing Medical University’s animal center under specific pathogen–free conditions with a 12-hour light/dark cycle. All experimental protocols received approval from Nanjing Medical University’s Institutional Animal Care and Use Committee. Mice with neutrophil-specific PAD4 knockout (PAD4^fl/fl^; S100A8-Cre, PAD4ΔPMN) were created by cross-breeding of PAD4^fl/fl^ mutant mice with MRP8-Cre [B6.Cg-Tg(S100A8-cre,-EGFP)1Ilw/J] mice. Heterozygous MRP8-Cre mice were crossed with homozygous PAD4^fl/fl^ mice to generate heterozygous MRP8-Cre/PAD4^fl/+^ progeny that were then crossed with PAD4^fl/fl^ mice. Of the resultant progeny, male MRP8-Cre/Padi4^fl/fl^ mice were used as the test group (referred to as neutrophil-specific PAD4^−/−^ [PAD4ΔPMN]). Male littermates that lacked MRP8-Cre were used as controls (PAD4^+/+^). C57BL/6 wild-type mice, PAD4^fl/fl^ C57BL/6 mice, and S100A8-Cre C57BL/6 mice were sourced from GemPharmatech Co. Ltd.

Male mice, 6–8 weeks old, were subjected to a 60% kcal high-fat diet (HFD) (Research Diets) for 12 weeks to model MASL. To model MASH, male mice were fed a methionine- and choline-deficient diet (MCD) (Research Diets) for 12 weeks. An additional MASH model was based on a prior study (54). For 12 weeks, mice were fed a Western diet (WD) with 21.1% fat, 41% sucrose, and 1.25% cholesterol (Teklad Diets), and a high-sugar solution (23.1 g/L d-fructose and 18.9 g/L d-glucose; Sigma-Aldrich). Mice received weekly intraperitoneal injections of 0.2 μL (0.32 μg)/g body weight of CCl_4_ (Sigma-Aldrich), starting with the diet initiation.

For neutrophil depletion, 25 μg/d of anti-Ly6G antibody (Bio X Cell) was administered intraperitoneally to mice during the latter part of the modeling period; control mice received an equivalent dose of IgG2a isotype (Bio X Cell).

### Single-cell RNA sequencing.

Liver single-cell sequencing data included a total of 6 MASL patients and 6 MASH patients, sourced from 2 different cohorts. The first cohort included liver biopsy samples collected from 3 MASL patients and 3 MASH patients, which were subjected to single-cell sequencing. The second cohort consisted of single-cell sequencing data acquired from the public database GSE159977, including 3 MASL patients and 3 MASH patients (55).

The fresh liver tissue was stored in GEXSCOPE Tissue Preservation Solution (Singleron) and transported to the Singleron laboratory on ice as soon as possible. The specimens were washed with Hanks balanced salt solution 3 times and minced into 1 to 2 mm pieces. Then the tissue pieces were digested with 2 mL GEXSCOPE Tissue Dissociation Solution (Singleron) at 37°C for 15 minutes in a 15 mL centrifuge tube with sustained agitation. After digestion, 40 μm sterile strainers were used to filter the samples, and the samples were centrifuged at 300*g* for 5 minutes. Then the supernatant was discarded, and the sediment was resuspended in 1 mL PBS (HyClone). To remove the red blood cells, 2 mL GEXSCOPE red blood cell lysis buffer (Singleron) was added at 25°C for 10 minutes. The solution was then centrifuged at 500*g* for 5 minutes and suspended in PBS. The sample was stained with trypan blue (Sigma-Aldrich) and microscopically evaluated.

Single-cell suspensions were converted to barcoded scRNA-Seq libraries using the Chromium Single Cell Library and Gel Bead & Multiplex Kit (10x Genomics) and following the manufacturer’s instructions. Briefly, cells were partitioned into Gel Beads in Emulsion in the Chromium Controller instrument where cell lysis and barcoded reverse transcription of RNA occurred. Libraries were prepared using 10x Genomics Library Kits and sequenced on Illumina Nova6000 with 150 bp paired-end reads.

### scRNA-Seq quantifications and statistical analysis.

Raw reads were processed to generate gene expression profiles using Cell Ranger v7.0.0. Reads from the 10x library were mapped to GRCh38 with Ensembl version 98 gene annotation. Reads with the same cell barcode, unique molecular identifier (UMI), and gene were grouped together to calculate the number of UMIs per gene per cell. The UMI count tables of each cellular barcode were used for further analysis.

### Statistics.

Data are presented as means ± SEM or as medians with range or quartile distributions for continuous variables. Categorical variables were represented by absolute numbers and percentages. The Shapiro-Wilk test assessed data normality, and Levene’s test evaluated variance homogeneity. For normally distributed data, a 2-tailed Student’s *t* test was used for comparisons between 2 groups. For comparisons among 3 or more groups, 1-way ANOVA was used. When data were not normally distributed, the Kruskal-Wallis test was used. The relationship between 2 variables was quantified using Pearson’s or Spearman’s rank correlation coefficient (ρ), based on data characteristics. Multiple comparisons used Dunn’s post hoc test with the Bonferroni method for error adjustment. To examine the correlation between neutrophil tertiles and liver histopathology, a linear-by-linear association in the χ^2^ test was used. A *P* value of less than 0.05 indicated statistical significance. All statistical analyses were performed using SPSS Statistics software version 26.0.

### Study approval.

The study was approved by the Ethics Committee of the First Affiliated Hospital of Nanjing Medical University (2017-SR-171-A2). All animal studies were performed following a protocol approved by Nanjing Medical University’s Institutional Animal Care and Use Committee.

### Data availability.

The data used to support the findings of this study are available within this article and within the Supporting Data Values file. The scRNA-seq data reported in this study have been deposited in the Genome Sequence Archive (GSA) at the National Genomics Data Center (NGDC), China National Center for Bioinformation/Beijing Institute of Genomics, Chinese Academy of Sciences (GSA-Human accession no. HRA013570) and are publicly accessible at https://ngdc.cncb.ac.cn/gsa-human Bulk RNA-Seq data were deposited in the NCBI’s Gene Expression Omnibus (GEO) database under accession GSE308064 (https://www.ncbi.nlm.nih.gov/geo/query/acc.cgi?acc=GSE308064). The mass spectrometry proteomics data were deposited to the ProteomeXchange Consortium via the iProX partner repository under identifiers PXD049272 and PXD049273 (http://proteomecentral.proteomexchange.org).

## Author contributions

JS, HZ, and HL developed the study concept. SH performed the computational analysis. JS, SH, SL, WG, and YS conducted experiments. JS, SH, and YG collected the clinical data. SL, WG, and YS managed the patients and assessed the clinical response. YW and MZ contributed biopsy samples and pathology analysis. QW, YS, and JS performed the single-cell RNA sequencing experiment and data analysis. JS and SH wrote the manuscript with the help of HL. All authors read and approved the final version of the manuscript.

## Funding support

- National Natural Science Foundation of China (grants 82103432 and 81800777).
- Medical Research Project of Jiangsu Commission of Health, China (H2023098).

## Supplementary Material

Supplemental data

Unedited blot and gel images

Supporting data values

## Figures and Tables

**Table 1:**
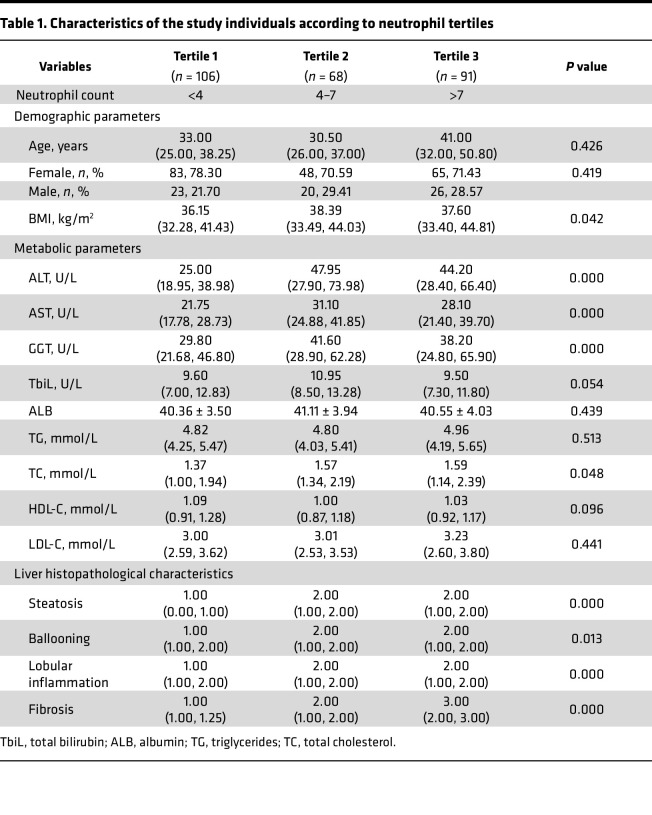
Characteristics of the study individuals according to neutrophil tertiles

**Table 2:**
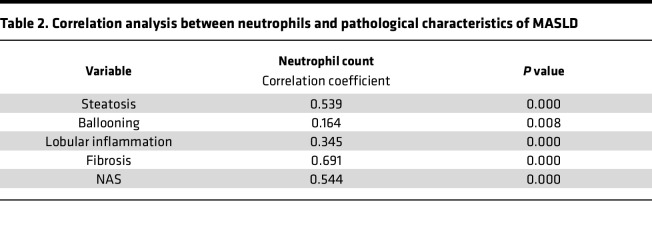
Correlation analysis between neutrophils and pathological characteristics of MASLD

## Version 1 01/09/2026

Electronic publication
