# Supplementary material for: PAD4+ neutrophils promote hepatic stellate cell activation and accelerate MASH fibrosis progression viaNET-DNA/TAOK1/MAPK pathways
Source: JCI Insight. 2026 Jan 9;11(1):e191479. doi: 10.1172/jci.insight.191479 (PMC12890522; doi:10.1172/jci.insight.191479)
Supplement: Supplemental data [file jciinsight-11-191479-s063.pdf]

**Supplementary materials and methods**

**Reagents**

The antibodies used were as follows:

For western blot and immunohistochemistry, rabbit monoclonal anti-MPO antibody (Abcam, Cambridge, UK), rabbit monoclonal anti-αSMA antibody (Abcam, Cambridge, UK), rabbit monoclonal anti-TIPM-1 antibody (Abcam, Cambridge, UK), rabbit monoclonal anti-COL1A1 antibody (Abcam, Cambridge, UK), rabbit monoclonal anti-RAS antibody (Cell Signaling Technology, MA, USA), rabbit monoclonal anti-bRAF antibody (Cell Signaling Technology, MA, USA), rabbit monoclonal anti- phospho-bRAF antibody (Cell Signaling Technology, MA, USA), rabbit monoclonal anti-cRAF antibody (Cell Signaling Technology, MA, USA), rabbit monoclonal anti- phospho-cRAF antibody (Cell Signaling Technology, MA, USA), rabbit monoclonal anti-MEK antibody (Cell Signaling Technology, MA, USA), rabbit monoclonal anti- phospho-MEK antibody (Cell Signaling Technology, MA, USA), rabbit monoclonal anti-JNK antibody (Cell Signaling Technology, MA, USA), rabbit monoclonal anti- phospho-JNK antibody (Cell Signaling Technology, MA, USA), rabbit monoclonal anti-ERK antibody (Cell Signaling Technology, MA, USA), rabbit monoclonal anti- phospho-ERK antibody (Cell Signaling Technology, MA, USA), rabbit monoclonal anti-p38 antibody (Cell Signaling Technology, MA, USA), rabbit monoclonal anti- phospho-p38 antibody (Cell Signaling Technology, MA, USA), rabbit monoclonal anti-GAPDH antibody (Cell Signaling Technology, MA, USA), mouse monoclonal anti-GAPDH antibody (Cell Signaling Technology, MA, USA), rabbit

monoclonal anti-TAOK1 antibody (Thermo Fisher Scientific, MA, USA) , Mouse monoclonal anti-MAPKAPK5 antibody (Thermo Fisher Scientific, MA, USA) were used were used (i.e., all antibodies used are listed in Supplemental Table 4).

For immunofluorescence, rabbit monoclonal anti-αSMA antibody (Abcam, Cambridge, UK), mouse monoclonal anti-MPO antibody (Abcam, Cambridge, UK), rabbit monoclonal anti-H3cit antibody (Abcam, Cambridge, UK), rabbit monoclonal anti-TAOK1 antibody (Thermo Fisher Scientific, MA, USA), Rhodamine Red-X (RRX) goat anti-mouse IgG (H+L) and FITC-AffiniPure goat anti-rabbit IgG (H+L) (Jackson, PA, USA) were used.

For flow cytometry, freshly isolated liver nonparenchymal cells（LNPCs) were incubated with mouse Fc receptor blocker to prevent non-specific binding. Then, the cells were incubated with various staining antibodies, including APC Cyanine7conjugated anti-mouse CD45 (clone 30F11, Miltenyi Research Inc. , CA, USA), PEvio770 conjugate anti-mouse CD11b (clone M1/70, Thermo Fisher Scientific, MA, USA), APC conjugated anti-mouse F4/80 (clone BM8, Thermo Fisher Scientific, MA, USA), FITC-conjugated anti-mouse Ly6C (clone AL21, BD Biosciences, NJ, USA), PE-conjugated anti-mouse Ly6G (clone RB6-8C5, Thermo Fisher Scientific, MA, USA). In identifying the proportion of $PAD4^+$ neutrophils, the antibodies used are as follows: Alexa Fluor® 488 monoclonal anti-rabbit PAD4 (Abcam, Cambridge, UK), APC conjugated anti-mouse CD45 (clone I3/2.3, Biolegend, CA, USA), PE-conjugated anti-mouse Ly6G (clone 1A8, Biolegend, CA, USA), PerCP/Cyanine5.5 anti-mouse CD11b (M1/70, Biolegend, CA, USA).

The following chemicals were also utilized: IL-8 (Sigma-Aldrich, MO, USA) , G-CSF (Sigma-Aldrich, MO, USA) , TNF-beta (Sigma-Aldrich, MO, USA), Myeloperoxidase Inhibitor PF1355 (Cayman Chemical, MI, USA), ELANE Inhibitor ONO5046 (Cayman Chemical, MI, USA), DNase I (Sigma-Aldrich, MO, USA), TAO Kinase inhibitor 1 (MedChemexpress, NJ, USA), MAPK Inhibitor U0126 (Sigma-Aldrich, MO, USA).

**Cell culture**

Human hepatic stellate cell line LX-2 was purchased from Procell Life Science & Technology (Wuhan, China) and cultured in the complete DMEM (ATCC, VA, USA) supplemented with 10% fetal bovine serum (Hyclone, UT, USA).

**Serum aspartate aminotransferase (AST) and alanine aminotransferase (ALT) analysis**

Blood was collected from the post-orbital venous plexus. The serum was separated by centrifugation and the levels of AST and ALT in the serum were measured with an automated chemical analyzer (MODULAR EVO 4200, Switzerland).

**Neutrophil isolation and in vitro NETs induction**

Neutrophils were isolated from peripheral blood of MASLD patients using Polymorphprep™ (Axis-Shield PoC AS, OSLO, Norway). The lysis of red blood cells was performed using a hypotonic solution (Solarbio, Beijing, China) according to the manuscript. The neutrophils ($5x10^6$ cells) seeded in 10cm culture plates were cultured in RPMI 1640 with 10% fetal bovine serum in a humidified 5% $CO_2$ incubator at 37°C. As determined by Wright staining and FACS analysis, the final neutrophil suspensions

contained fewer than 0.1% monocytes or lymphocytes. Neutrophil viability exceeded 98% after up to 6h in culture, as determined by trypan blue exclusion and by Annexin V/propidium iodide FACS analysis. Isolated neutrophils were incubated with NETs inducers (100ng/ml IL-8, 100ng/ml G-CSF, 100ng/ml TNF-beta). Following stimulation for 6h, the supernatant was carefully discarded to remove any soluble factors released into the media. The cell culture wells were then gently washed with 2 ml of cold PBS to collect the NETs structures that had adhered to the bottom surface. The PBS containing the washed-off material was then centrifuged at 1000g for 10 minutes at 4°C, and the cell-free supernatant was collected as the NET preparation.

**Detection of supernatant NETs**

NETs in supernatant were assayed by SYTOX Green fluorescence. SYTOX Green is a membrane-impermeable DNA-binding dye that can be used to quantify NETs-DNA. At the end of the incubation with NETs inducers, neutrophils were incubated with SYTOX Green (5μM) for 30min at 37°C. Then, after washing with PBS, NETs were observed by fluorescence microscopy.

**Purification of NETs-DNA**

The NETs, previously separated, were subjected to fragmentation using a sonicator, achieving fragment sizes ranging from 200 to 500 base pairs. Following this, the DNA from these NETs was extracted and purified utilizing the MicroElute DNA Clean Up Kit (OMEGA, GA, USA).

**Isolation of cell membrane protein**

Cell membrane protein of LX-2 was isolated with Mem-PER™ Plus membrane

protein extraction kit (Thermo Fisher Scientific, MA, USA). The procedures were according to the manufacturer's recommendation.

**DNA-pull down**

NETs-DNA was biotinylated with Biotin 3' End DNA Labelling Kit (Thermo Fisher Scientific, MA, USA) according to the manufacturer's instructions. Membrane protein of LX-2 was co-cultured with 500 ng of NETs-DNA, which had been biotinylated. This reaction took place in a 400 μl solution of IP lysis buffer (Thermo Fisher Scientific, MA, USA), and the mixture was maintained at ambient temperature for a duration of one hour. Subsequent to this, the resulting complex of protein and DNA was further treated with 50 μl of streptavidin-agarose beads, also at room temperature, for an additional hour. Following this incubation period, the beads were subjected to a triple washing using the IP lysis buffer. The next phase involved the separation of these beads via gradient gel electrophoresis. The separation process was followed by western blot to identify specific protein bands. Lastly, these identified bands were subjected to a detailed analysis using mass spectrometry for precise identification.

**RNA sequencing analysis (RNA-seq)**

RNA was extracted and subsequently converted into cDNA for the creation of a sequenced library compatible with Illumina indices. The sequencing was conducted at the Beijing Genomics Institute in China, utilizing the BGISEQ-500 system. A gene was considered to be significantly differentially expressed if it exhibited a more than twofold change in expression compared to the control, coupled with a $p$ value below 0.05 after adjustment. Gene Ontology was employed to analyze the heat map, with the

Cluster software for analysis and Java Treeview for visualization purposes. The investigation of differentially expressed genes (DEGs) involved the use of Gene Ontology tools, specifically AMIGO and DAVID. Additionally, the Kyoto Encyclopedia of Genes and Genomes annotations was utilized to assess the enrichment levels of these DEGs.

**Bone Marrow-Derived Neutrophil (BMDN) Culture and Adoptive Transfer**

Bone marrow cells were harvested from the femurs and tibias of mice by flushing with PBS using 25-gauge needles. The resulting cell suspension was gently dissociated and passed through a 40 μm cell strainer to obtain a single-cell suspension. Red blood cells were lysed using RBC lysis buffer. To block GM-CSF signaling, anti-mouse GM-CSF antibody (BioLegend, #505401) was added to the culture. Neutrophils were then purified using the MojoSort™ Mouse Ly6G Selection Kit (BioLegend, #480124) and designated as bone marrow-derived neutrophils (BMDNs) for downstream applications.

For adoptive transfer experiments, purified BMDNs from PAD4$^{+/+}$ and PAD4ΔPMN mice were counted using Trypan Blue exclusion and resuspended at a concentration of $1\times10^8$ viable cells/mL. Recipient mice were intravenously injected with $1\times10^7$ BMDNs (in 100 μL PBS) per mouse at designated time points. Starting from week 6 of the WD/$CCl_4$ dietary model, BMDNs were administered weekly via tail vein injection. For leukocyte quantification, peripheral blood was collected into EDTA-containing tubes, and leukocytes were stained and analyzed by flow cytometry.

**Western blot assay**

The expression of the indicated proteins was assayed using western blot. The indicated molecules for the analyses were listed in the Reagents. The relative levels were normalized against GAPDH in the same samples.

**Enzyme-linked immunosorbent assay**

ELISA (Invitrogen, CA, USA) was applied to measure CXCL1, CXCL2, CXCL3, CXCL5, CXCL6 and CXCL8 levels in the liver of MASL and MASH patients. Each experiment was repeated in triplicates.

**Luminex Assay**

Using Luminex technology, we analyzed the expression of various factors in liver samples. For every 10 mg of tissue, 200 μL of pre-cooled lysis buffer was added. The tissue was homogenized using a tissue homogenizer, and the supernatant was collected after centrifugation. According to the instructions of the kit (Bio-Rad Laboratories, CA, USA), beads, standards, quality controls, and samples were sequentially added in volumes of 25-50 μL and incubated at room temperature at 800 rpm for 0.5-1 hour or overnight at 4°C. Beads were then washed three times with 100-200 μL of wash buffer. Next, 25-50 μL of detection antibodies were added and incubated at room temperature at 800 rpm for 0.5-1 hour. The beads were washed again three times with 100-200 μL of wash buffer, followed by the addition of 50 μL of PE-streptavidin and incubation at room temperature at 800 rpm for 10-30 minutes. After three more washes with 100-200 μL of wash buffer, 100-150 μL of sheath fluid/wash buffer was added and incubated at room temperature at 800 rpm for 0.5-2 minutes before proceeding with the analysis on the machine.

**Immunofluorescence**

Samples embedded in paraffin were sectioned to a thickness of 4 micrometers. Antigen retrieval was performed in a pressure cooker using 0.01M citrate buffer for a duration of 20 minutes. Subsequently, the sections were subjected to a blocking process using PBS supplemented with 10% bovine serum albumin at ambient temperature for two hours. Following the blocking step, the samples were incubated with primary antibodies at 4 ℃ throughout the night. This was followed by a one-hour room temperature incubation with Rhodamine Red-X (RRX) goat anti-mouse IgG (H+L) and FITC-AffiniPure goat anti-rabbit IgG (H+L) (Jackson ImmunoResearch Laboratories, PA, USA). For nuclear staining, DAPI (Sigma-Aldrich, MO, USA) was employed. Confocal microscopy images were captured using the Zeiss LSM510 system (Oberkochen, Germany).

**Immunohistochemistry**

The expression of MPO in the liver was detected by immunohistochemistry (IHC). IHC was applied on paraffin-embedded formalin-fixed tissue samples according to standard protocols.

**LC-MS/MS analysis**

In this analysis, tryptic peptides were reconstituted in solvent A, consisting of 0.1% formic acid, and then applied to a custom-made analytical column with a reversed-phase configuration (75 μm internal diameter, 15 cm length). We implemented a gradient elution program using solvent B (0.1% formic acid in 98% acetonitrile) that gradually increased from 6% to 23% over 26 minutes, followed by a rise to 35% in the

next 8 minutes, and finally peaking at 80% for the concluding 3 minutes. This elution was carried out at a steady flow rate of 400 nL/min using an EASY-nLC 1000 UPLC system. Post-column, the peptides were introduced to a nano-electrospray ionization (NSI) source and then analyzed via tandem mass spectrometry on a Q ExactiveTM Plus (Thermo) system, integrated online with the UPLC. An electrospray voltage of 2.0 kV was applied. The mass-to-charge (m/z) scan range spanned from 350 to 1800 for a comprehensive scan, capturing intact peptides in the Orbitrap at a resolution of 70,000. For MS/MS, peptides were selected under a normalized collision energy (NCE) setting of 28, with their fragments detected at a 17,500 resolution in the Orbitrap. The system followed a data-dependent approach alternating between a singular MS scan and 20 MS/MS scans, incorporating a 15.0 s dynamic exclusion period. The automatic gain control (AGC) threshold was set to 5E4, and the fixed first mass was established at 100 m/z.

**Flow cytometry**

Isolated liver non-parenchymal cells (LNPCs) initially underwent treatment with a blocker for mouse Fc receptors to mitigate unspecific bindings. Subsequently, these cells were exposed to a range of fluorescently labeled antibodies for staining. The antibodies used were listed in the Reagents.

**scRNA-seq quality control, dimension-reduction and clustering (Scanpy)**

Scanpy v1.8.1 was used for quality control, dimensionality reduction and clustering under Python 3.7. For each sample dataset, we filtered expression matrix by the following criteria: 1) cells with gene count less than 200 or with top 2% gene count

were excluded; 2) cells with top 2% UMI count were excluded; 3) cells with mitochondrial content ＞ 30% were excluded; 4) genes expressed in less than 5 cells were excluded. After filtering, 67763 cells were retained for the downstream analyses, with on average 1381.502 genes and 3938.929 UMIs per cell. The raw count matrix was normalized by total counts per cell and logarithmically transformed into normalized data matrix. Top 2000 variable genes were selected by setting flavor = 'seurat'. Principal Component Analysis (PCA) was performed on the scaled variable gene matrix, and top 20 principal components were used for clustering and dimensional reduction. Cells were separated into 25 clusters by using Louvain algorithm and setting resolution parameter at 1.2. Cell clusters were visualized by using Uniform Manifold Approximation and Projection (UMAP) (1).

**Batch Effect removal**

Harmony: Batch effect between samples was removed by Harmony v1.0 using the top 20 principal components from PCA (2).

**Differentially expressed genes (DEGs) analysis (scanpy)**

To identify differentially expressed genes (DEGs), we used the scanpy.tl.rank_genes_groups function based on Wilcoxon rank sum test with default parameters, and selected the genes expressed in more than 10% of the cells in either of the compared groups of cells and with an average log(Fold Change) value greater than 0.25 as DEGs. Adjusted p value was calculated by benjamini-hochberg correction and the value 0.05 was used as the criterion to evaluate the statistical significance.

**Pathway enrichment analysis**

To investigate the potential functions of DEGs between neutrophils in MASH and MASL, Gene Ontology (GO) and Kyoto Encyclopedia of Genes and Genomes (KEGG) analysis were used with the “clusterProfiler” R package v 4.0.2 (3). Pathways with p_adj value less than 0.05 were considered as significantly enriched. Selected significant pathways were plotted as lollyplot. GSEA was performed on Neutrophils' DEGs between MASH and MASL. For GSVA pathway enrichment analysis, the average gene expression of each cell type was used as input data (4). Gene Ontology gene sets including molecular function (MF), biological process (BP), and cellular component (CC) categories were used.

**Celltype annotation, Cell-type recognition with Cell-ID**

Cell-ID is multivariate approach that extracts gene signatures for each individual cell and perform cell identity recognition using hypergeometric tests (HGT). Dimensionality reduction was performed on normalized gene expression matrix through multiple correspondence analysis, where both cells and genes were projected in the same low dimensional space. Then a gene ranking was calculated for each cell to obtain most featured gene sets of that cell. HGT were performed on these gene sets against liver reference from SynEcoSys database, which contains all cell-type's featured genes. Identity of each cell was determined as the cell-type has the minimal HGT p value. For cluster annotation, Frequency of each cell-type was calculated in each cluster, and cell-type with highest frequency was chosen as cluster’s identity (5).

The cell type identification of each cluster was determined according to the expression of canonical markers from the reference database SynEcoSys$^{TM}$ (Singleron

Biotechnology). SynEcoSys$^{TM}$ contains collections of canonical cell type markers for single-cell seq data, from CellMakerDB, PanglaoDB and recently published literatures.

**Subtyping of major cell types**

To obtain a high-resolution map of MPs, T and NK, Neutrophils cells, the specific cluster were extracted and reclustered for more detailed analysis following the same procedures described above and by setting the clustering resolution as 1.2, 1.2, 0.2 respectively.

**Cell-cell interaction analysis: CellPhoneDB**

Cell-cell interaction (CCI) between Neutrophils and other cell types were predicted based on known ligand–receptor pairs by Cellphone DB (v 4.0.0) version (6). Permutation number for calculating the null distribution of average ligand-receptor pair expression in randomized cell identities was set to 1000. Individual ligand or receptor expression was thresholded by a cutoff based on the average log gene expression distribution for all genes across each cell type. Predicted interaction pairs with p value <0.05 and of average log expression > 0.1 were considered as significant and visualized by heatmap_plot and dot_plot in CellphoneDB.

**Pseudotime Trajectory Analysis: monocle2**

Cell differentiation trajectory of monocyte subtypes was reconstructed with the Monocle2 v 2.10.0 (7). For constructing the trajectory, top 2000 highly variable genes were selected by Seurat (v3.1.2) FindVairableFeatures, and dimension-reduction was performed by DDRTree. The trajectory was visualized by plot_cell_trajectory function in Monocle2.

**UCell Gene Set Scoring**

Gene set scoring was performed using the R package UCell v 2.2.0 (8). UCell scores are based on the Mann-Whitney U statistic by ranking query genes in order of their expression levels in individual cells. Because UCell is a rank-based scoring method, it is suitable to be used in large datasets containing multiple samples and batches.

The NETs formation gene signature was defined based on previously published NETs-associated gene sets(9). The signature includes canonical NETs-related genes such as: PAD4, ELANE, MPO, H3F3B, TLR9, CTSG, LTF, GSDMD, and others involved in chromatin decondensation and NETs extrusion.

For analysis, we used: Seurat (v4.3) AddModuleScore function to calculate NETs gene signature scores in each neutrophil cluster (scRNA-seq data). Scores were compared between groups using Wilcoxon rank-sum test. Visualization in dot plots shows differential NETs scores across neutrophil subsets.

**scGSVA**

To do GSVA analysis for single cell data, we use scGSVA (https://github.com/guokai8/scGSVA), which make use of ssgsea methods to score individual cells respectively to generate multiple pathway enrichment score matrices. Using package of Limma to calculate the differential enrichment score for pathway, which absolute value of t greater than 1.96 as significant difference, among cell types.

**Transcription factor regulatory network analysis (pySCENIC)**

Transcription factor network was constructed by pyscenic (v0.11.0) using scRNA

expression matrix and transcription factors in AnimalTFDB (10). First, GRNBoost2 predicted a regulatory network based on the co-expression of regulators and targets. CisTarget was then applied to exclude indirect targets and to search transcription factor binding motifs. After that, AUCell was used for regulon activity quantification for every cell. Cluster-specific TF regulons were identified according to Regulon Specificity Scores (RSS) and the activity of these TF regulons were visualized in heatmaps.

Reference：


1. Wolf FA, Angerer P, and Theis FJ. SCANPY: large-scale single-cell gene expression data analysis. *Genome Biol.* 2018;19(1):15.
2. Butler A, Hoffman P, Smibert P, Papalexi E, and Satija R. Integrating single-cell transcriptomic data across different conditions, technologies, and species. *Nat Biotechnol.* 2018;36(5):411-20.
3. Yu G, Wang LG, Han Y, and He QY. clusterProfiler: an R package for comparing biological themes among gene clusters. *OMICS.* 2012;16(5):284-7.
4. Hanzelmann S, Castelo R, and Guinney J. GSVA: gene set variation analysis for microarray and RNA-seq data. *BMC Bioinformatics.* 2013;14:7.
5. Cortal A, Martignetti L, Six E, and Rausell A. Gene signature extraction and cell identity recognition at the single-cell level with Cell-ID. *Nat Biotechnol.* 2021;39(9):1095-102.
6. Efremova M, Vento-Tormo M, Teichmann SA, and Vento-Tormo R. CellPhoneDB: inferring cell-cell communication from combined expression of multi-subunit ligand-receptor complexes. *Nat Protoc.* 2020;15(4):1484-506.
7. Qiu X, Hill A, Packer J, Lin D, Ma YA, and Trapnell C. Single-cell mRNA quantification and differential analysis with Census. *Nat Methods.* 2017;14(3):309-15.
8. Andreatta M, and Carmona SJ. UCell: Robust and scalable single-cell gene signature scoring. *Comput Struct Biotechnol J.* 2021;19:3796-8.
9. Papayannopoulos V. Neutrophil extracellular traps in immunity and disease. *Nat Rev Immunol.* 2018;18(2):134-47.
10. Van de Sande B, Flerin C, Davie K, De Waegeneer M, Hulselmans G, Aibar S, et al. A scalable SCENIC workflow for single-cell gene regulatory network analysis. *Nat Protoc.* 2020;15(7):2247-76.

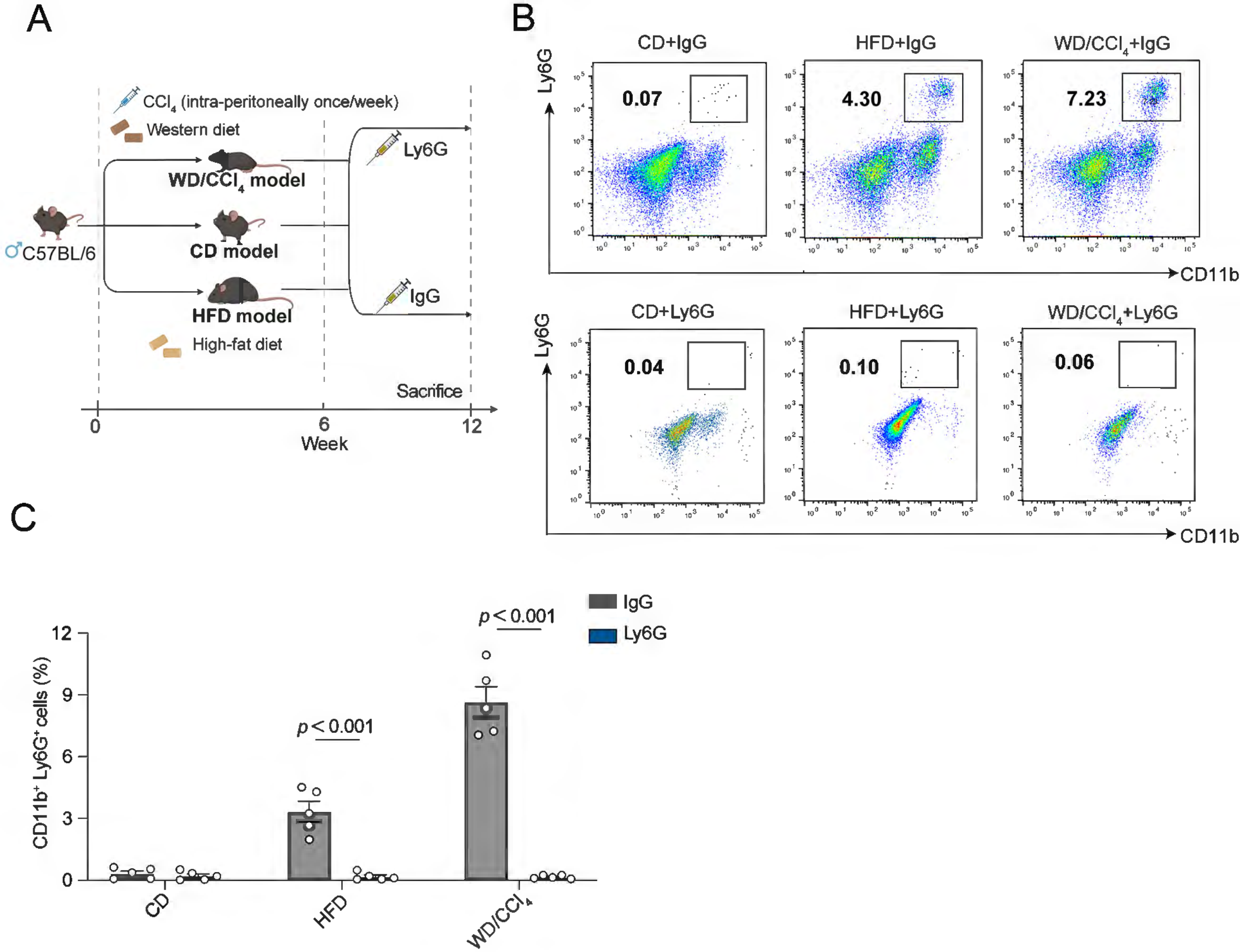

A
CCl4 (intra-peritoneally once/week)
Western diet
WD/CCl4 model
CD model
HFD model
High-fat diet
♂C57BL/6
Ly6G
IgG
Sacrifice
0
6
12
Week
B
CD+IgG
HFD+IgG
WD/CCl4+IgG
0.07
4.30
7.23
CD+Ly6G
HFD+Ly6G
WD/CCl4+Ly6G
0.04
0.10
0.06
Ly6G
CD11b
C
CD11b+ Ly6G+ cells (%)
IgG
Ly6G
p < 0.001
p < 0.001
CD
HFD
WD/CCl4

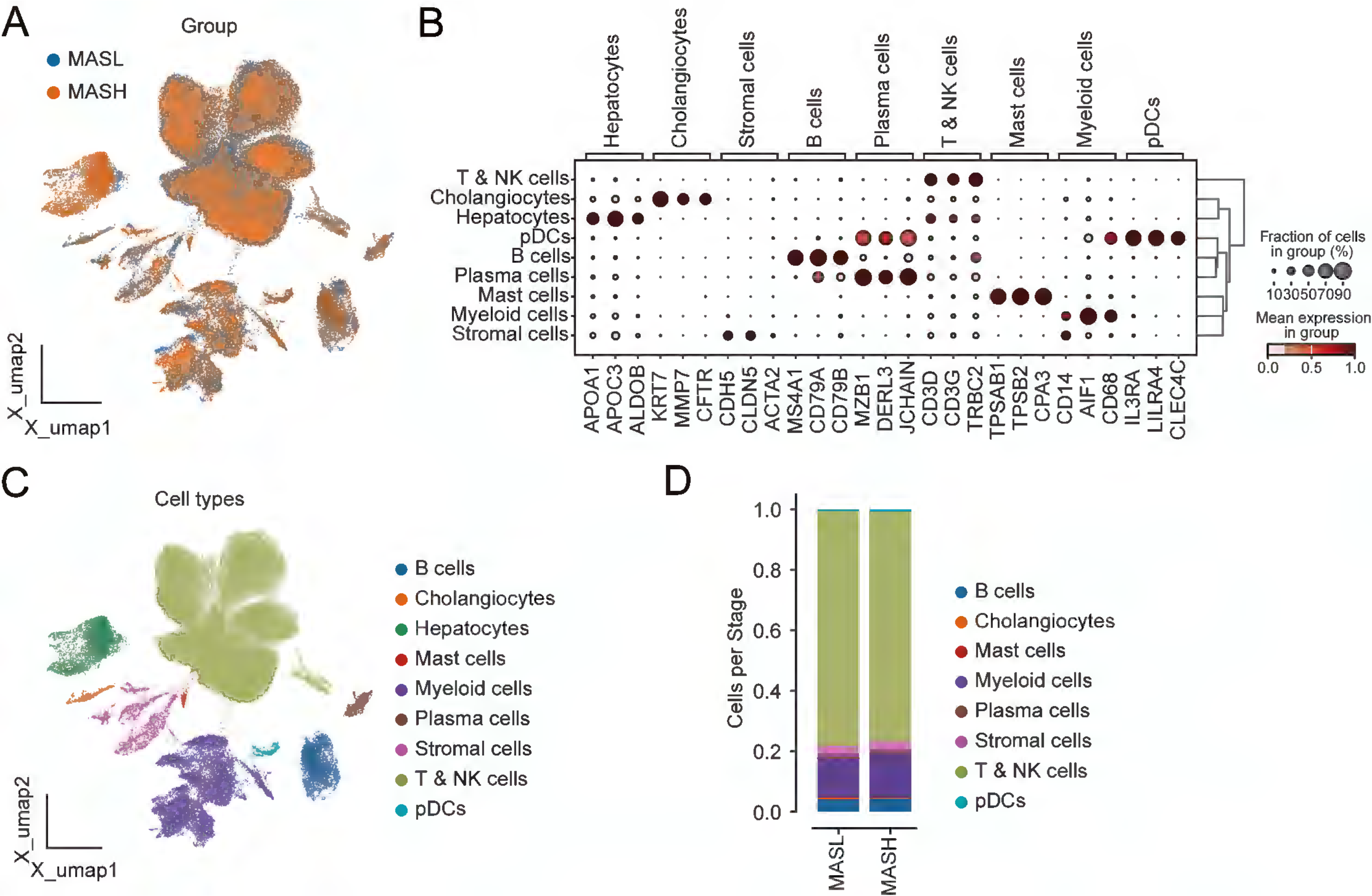

A
Group
MASL
MASH
X_umap2
X_umap1
B
Hepatocytes
Cholangiocytes
Stromal cells
B cells
Plasma cells
T & NK cells
Mast cells
Myeloid cells
pDCs
T & NK cells
Cholangiocytes
Hepatocytes
pDCs
B cells
Plasma cells
Mast cells
Myeloid cells
Stromal cells
APOA1
APOC3
ALDOB
KRT7
MMP7
CFTR
CDH5
CLDN5
ACTA2
MS4A1
CD79A
CD79B
MZB1
DERL3
JCHAIN
CD3D
CD3G
TRBC2
TPSAB1
TPSB2
CPA3
CD14
AIF1
CD68
IL3RA
LILRA4
CLEC4C
Fraction of cells in group (%)
1030507090
Mean expression in group
0.0
0.5
1.0
C
Cell types
B cells
Cholangiocytes
Hepatocytes
Mast cells
Myeloid cells
Plasma cells
Stromal cells
T & NK cells
pDCs
X_umap2
X_umap1
D
Cells per Stage
1.0
0.8
0.6
0.4
0.2
0.0
MASL
MASH
B cells
Cholangiocytes
Mast cells
Myeloid cells
Plasma cells
Stromal cells
T & NK cells
pDCs

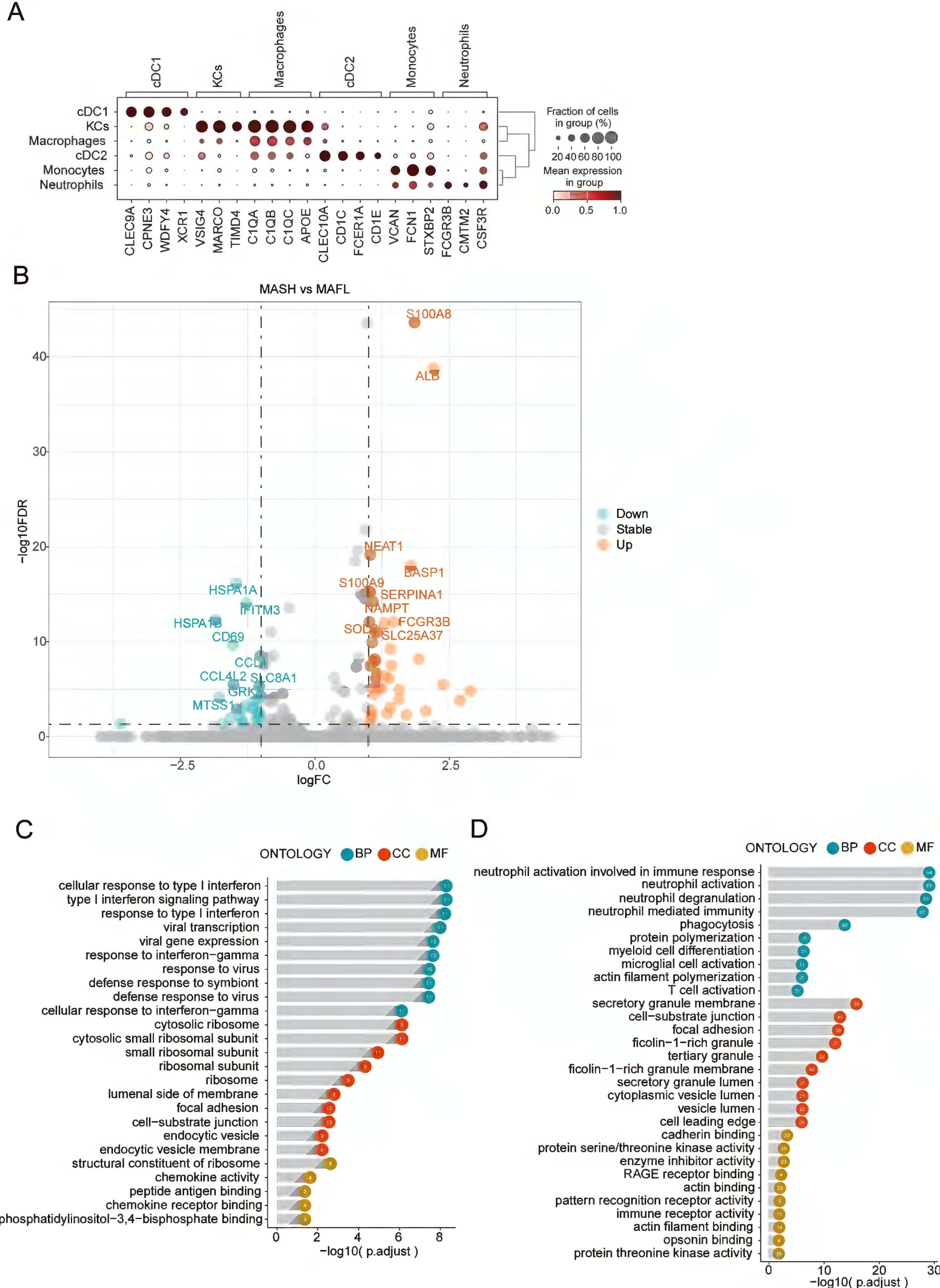

A
cDC1
KCs
Macrophages
cDC2
Monocytes
Neutrophils
CLEC9A CPNE3 WDFY4 XCR1 VSIG4 MARCO TIMD4 C1QA C1QB C1QC APOE CLEC10A CD1C FCER1A CD1E VCAN FCN1 STXBP2 FCGR3B CMTM2 CSF3R
Fraction of cells in group (%)
20 40 60 80 100
Mean expression in group
0.0 0.5 1.0
B
MASH vs MAFL
−log10FDR
logFC
S100A8
ALB
NEAT1
BASP1
S100A9
SERPINA1
NAMPT
FCGR3B
SLC25A37
HSPA1A
IFITM3
HSPA1B
CD69
CCL4L2
SLC8A1
MTSS1
Down
Stable
Up
C
ONTOLOGY BP CC MF
cellular response to type I interferon
type I interferon signaling pathway
response to type I interferon
viral transcription
viral gene expression
response to interferon−gamma
response to virus
defense response to symbiont
defense response to virus
cellular response to interferon−gamma
cytosolic ribosome
cytosolic small ribosomal subunit
small ribosomal subunit
ribosomal subunit
ribosome
lumenal side of membrane
focal adhesion
cell−substrate junction
endocytic vesicle
endocytic vesicle membrane
structural constituent of ribosome
chemokine activity
peptide antigen binding
chemokine receptor binding
phosphatidylinositol−3,4−bisphosphate binding
−log10( p.adjust )
D
ONTOLOGY BP CC MF
neutrophil activation involved in immune response
neutrophil activation
neutrophil degranulation
neutrophil mediated immunity
phagocytosis
protein polymerization
myeloid cell differentiation
microglial cell activation
actin filament polymerization
T cell activation
secretory granule membrane
cell−substrate junction
focal adhesion
ficolin−1−rich granule
tertiary granule
ficolin−1−rich granule membrane
secretory granule lumen
cytoplasmic vesicle lumen
vesicle lumen
cell leading edge
cadherin binding
protein serine/threonine kinase activity
enzyme inhibitor activity
RAGE receptor binding
actin binding
pattern recognition receptor activity
immune receptor activity
actin filament binding
opsonin binding
protein threonine kinase activity
−log10( p.adjust )

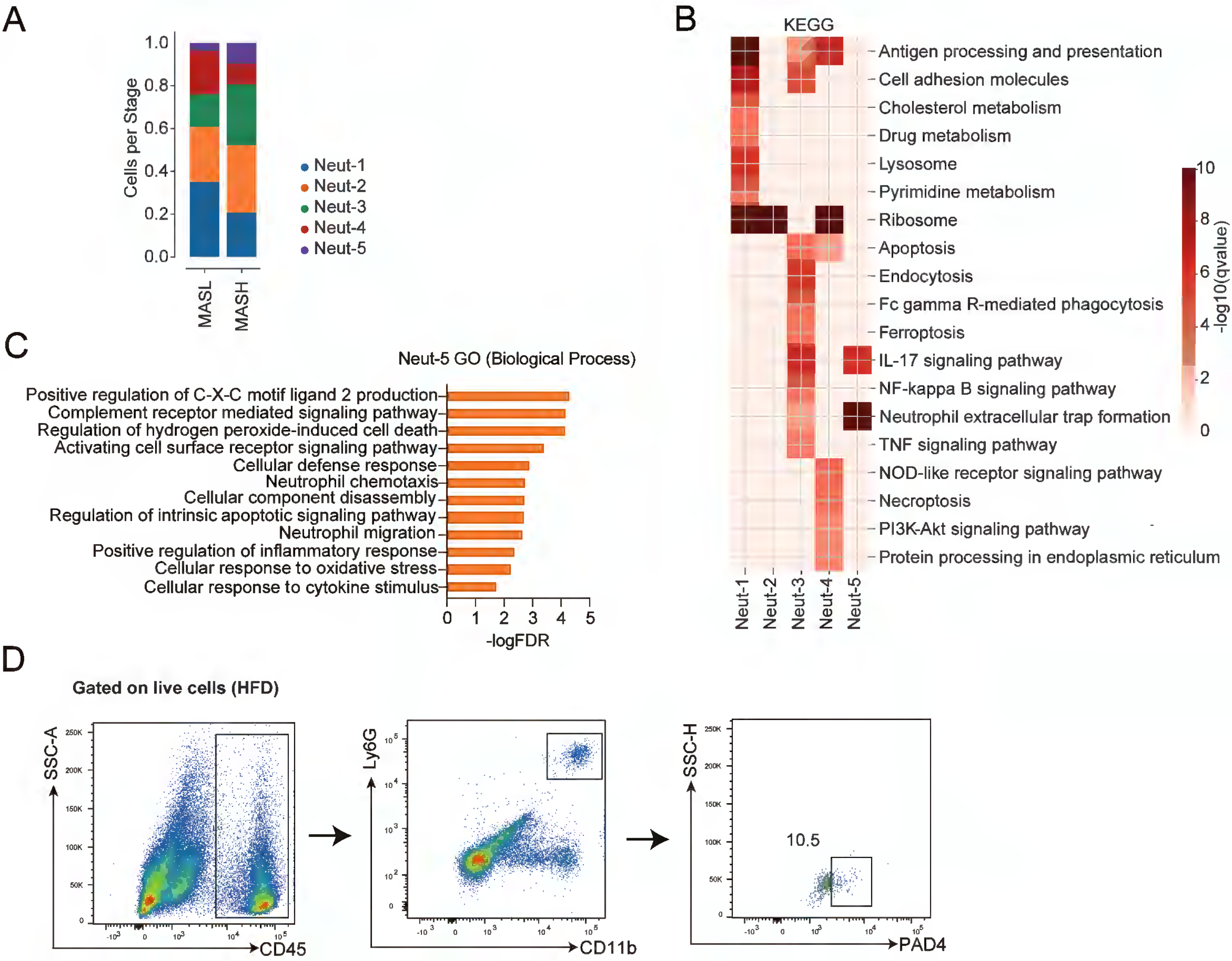

A
Cells per Stage
1.0
0.8
0.6
0.4
0.2
0.0
MASL
MASH
Neut-1
Neut-2
Neut-3
Neut-4
Neut-5
B
KEGG
Antigen processing and presentation
Cell adhesion molecules
Cholesterol metabolism
Drug metabolism
Lysosome
Pyrimidine metabolism
Ribosome
Apoptosis
Endocytosis
Fc gamma R-mediated phagocytosis
Ferroptosis
IL-17 signaling pathway
NF-kappa B signaling pathway
Neutrophil extracellular trap formation
TNF signaling pathway
NOD-like receptor signaling pathway
Necroptosis
PI3K-Akt signaling pathway
Protein processing in endoplasmic reticulum
Neut-1
Neut-2
Neut-3
Neut-4
Neut-5
-log10(qvalue)
10
8
6
4
2
0
C
Neut-5 GO (Biological Process)
Positive regulation of C-X-C motif ligand 2 production
Complement receptor mediated signaling pathway
Regulation of hydrogen peroxide-induced cell death
Activating cell surface receptor signaling pathway
Cellular defense response
Neutrophil chemotaxis
Cellular component disassembly
Regulation of intrinsic apoptotic signaling pathway
Neutrophil migration
Positive regulation of inflammatory response
Cellular response to oxidative stress
Cellular response to cytokine stimulus
0 1 2 3 4 5
-logFDR
D
Gated on live cells (HFD)
SSC-A
250K
200K
150K
100K
50K
0
CD45
Ly6G
CD11b
SSC-H
10.5
PAD4

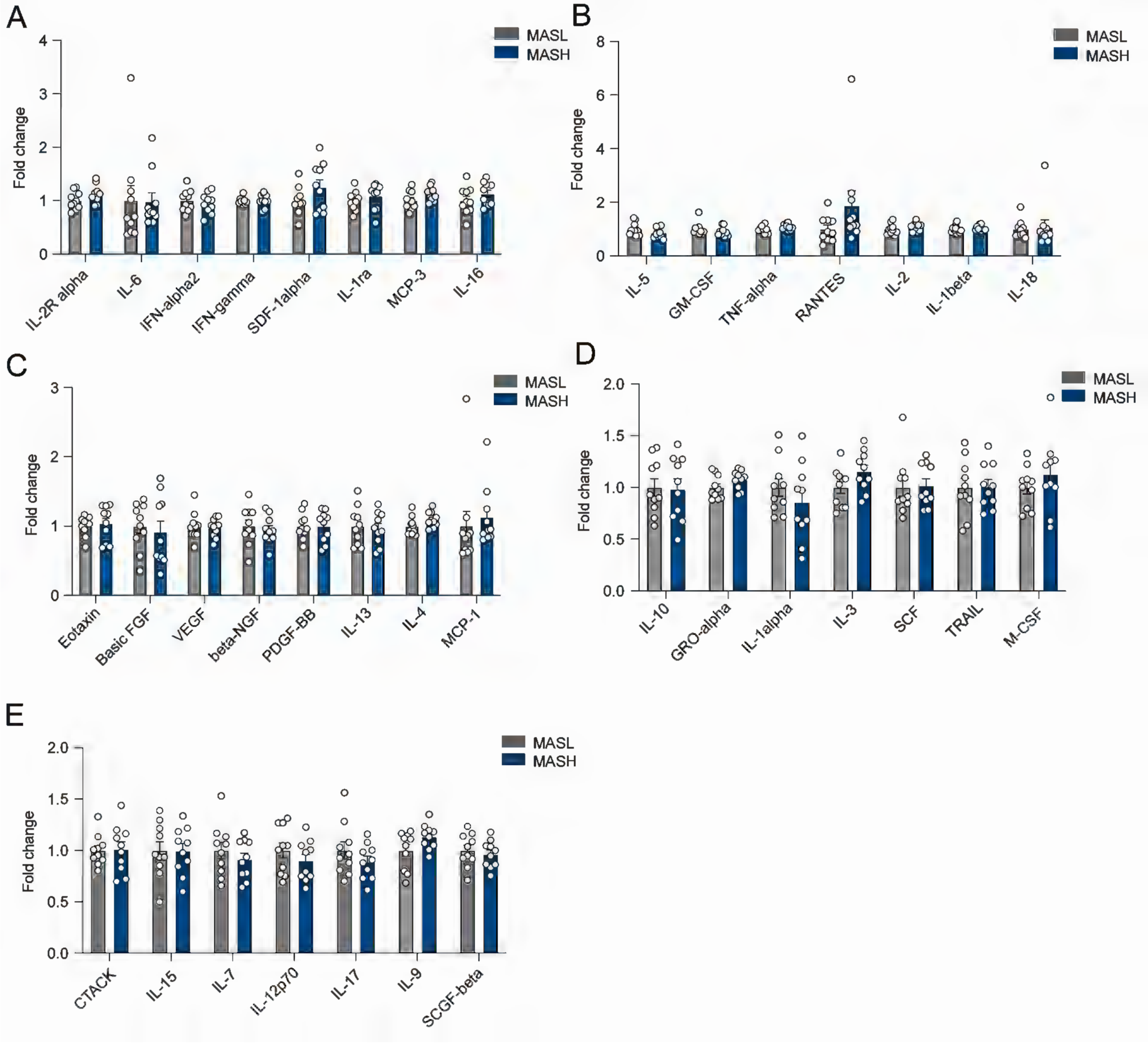
A
MASL
MASH
Fold change
4
3
2
1
0
IL-2R alpha
IL-6
IFN-alpha2
IFN-gamma
SDF-1alpha
IL-1ra
MCP-3
IL-16
B
MASL
MASH
Fold change
8
6
4
2
0
IL-5
GM-CSF
TNF-alpha
RANTES
IL-2
IL-1beta
IL-18
C
MASL
MASH
Fold change
3
2
1
0
Eotaxin
Basic FGF
VEGF
beta-NGF
PDGF-BB
IL-13
IL-4
MCP-1
D
MASL
MASH
Fold change
2.0
1.5
1.0
0.5
0.0
IL-10
GRO-alpha
IL-1alpha
IL-3
SCF
TRAIL
M-CSF
E
MASL
MASH
Fold change
2.0
1.5
1.0
0.5
0.0
CTACK
IL-15
IL-7
IL-12p70
IL-17
IL-9
SCGF-beta

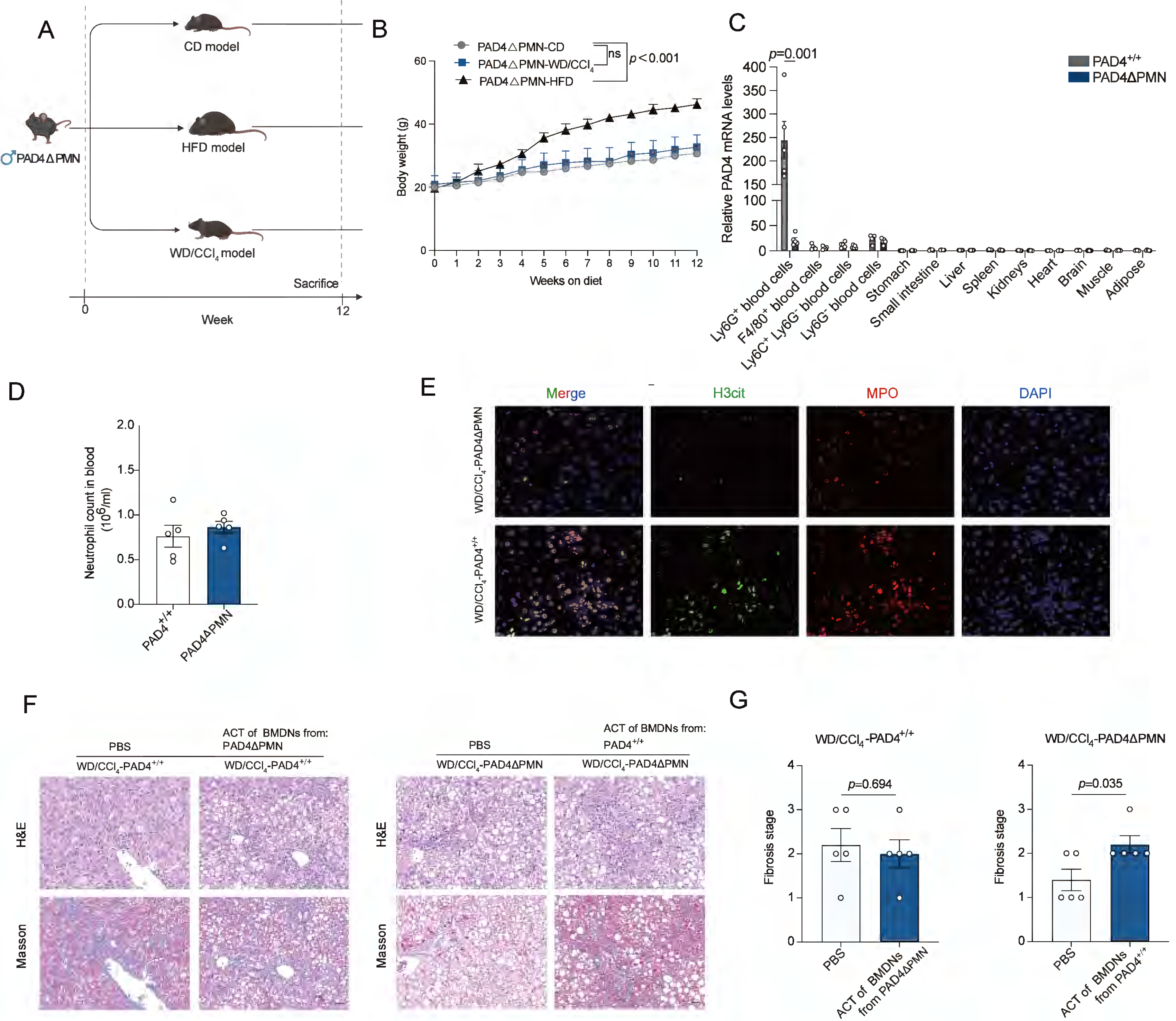

A
CD model
HFD model
WD/CCl4 model
♂PAD4ΔPMN
Sacrifice
0
12
Week
B
PAD4△PMN-CD
PAD4△PMN-WD/CCl4
PAD4△PMN-HFD
ns
p<0.001
Body weight (g)
Weeks on diet
C
p=0.001
PAD4+/+
PAD4ΔPMN
Relative PAD4 mRNA levels
Ly6G+ blood cells
F4/80+ blood cells
Ly6C+ Ly6G- blood cells
Ly6G- blood cells
Stomach
Small intestine
Liver
Spleen
Kidneys
Heart
Brain
Muscle
Adipose
D
Neutrophil count in blood (10^6/ml)
PAD4+/+
PAD4ΔPMN
E
Merge
H3cit
MPO
DAPI
WD/CCl4-PAD4ΔPMN
WD/CCl4-PAD4+/+
F
PBS
ACT of BMDNs from: PAD4ΔPMN
WD/CCl4-PAD4+/+
WD/CCl4-PAD4+/+
PBS
ACT of BMDNs from: PAD4+/+
WD/CCl4-PAD4ΔPMN
WD/CCl4-PAD4ΔPMN
H&E
Masson
G
WD/CCl4-PAD4+/+
p=0.694
Fibrosis stage
PBS
ACT of BMDNs from PAD4ΔPMN
WD/CCl4-PAD4ΔPMN
p=0.035
PBS
ACT of BMDNs from PAD4+/+

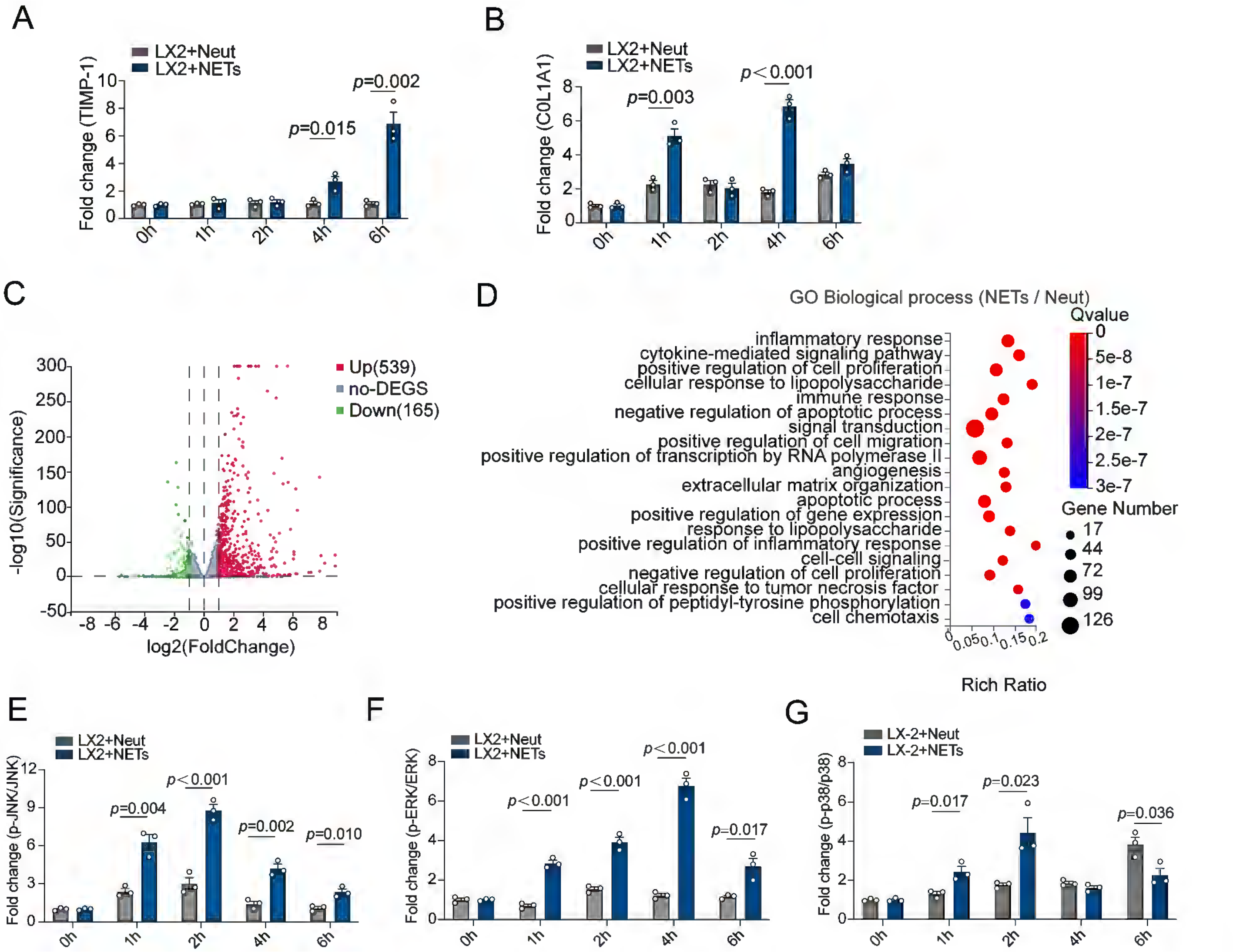

A
LX2+Neut
LX2+NETs
Fold change (TIMP-1)
p=0.015
p=0.002
0h
1h
2h
4h
6h
B
LX2+Neut
LX2+NETs
Fold change (COL1A1)
p=0.003
p<0.001
C
Up(539)
no-DEGS
Down(165)
-log10(Significance)
log2(FoldChange)
D
GO Biological process (NETs / Neut)
inflammatory response
cytokine-mediated signaling pathway
positive regulation of cell proliferation
cellular response to lipopolysaccharide
immune response
negative regulation of apoptotic process
signal transduction
positive regulation of cell migration
positive regulation of transcription by RNA polymerase II
angiogenesis
extracellular matrix organization
apoptotic process
positive regulation of gene expression
response to lipopolysaccharide
positive regulation of inflammatory response
cell-cell signaling
negative regulation of cell proliferation
cellular response to tumor necrosis factor
positive regulation of peptidyl-tyrosine phosphorylation
cell chemotaxis
Qvalue
Gene Number
Rich Ratio
E
LX2+Neut
LX2+NETs
Fold change (p-JNK/JNK)
p=0.004
p<0.001
p=0.002
p=0.010
F
LX2+Neut
LX2+NETs
Fold change (p-ERK/ERK)
p<0.001
p<0.001
p<0.001
p=0.017
G
LX-2+Neut
LX-2+NETs
Fold change (p-p38/p38)
p=0.017
p=0.023
p=0.036

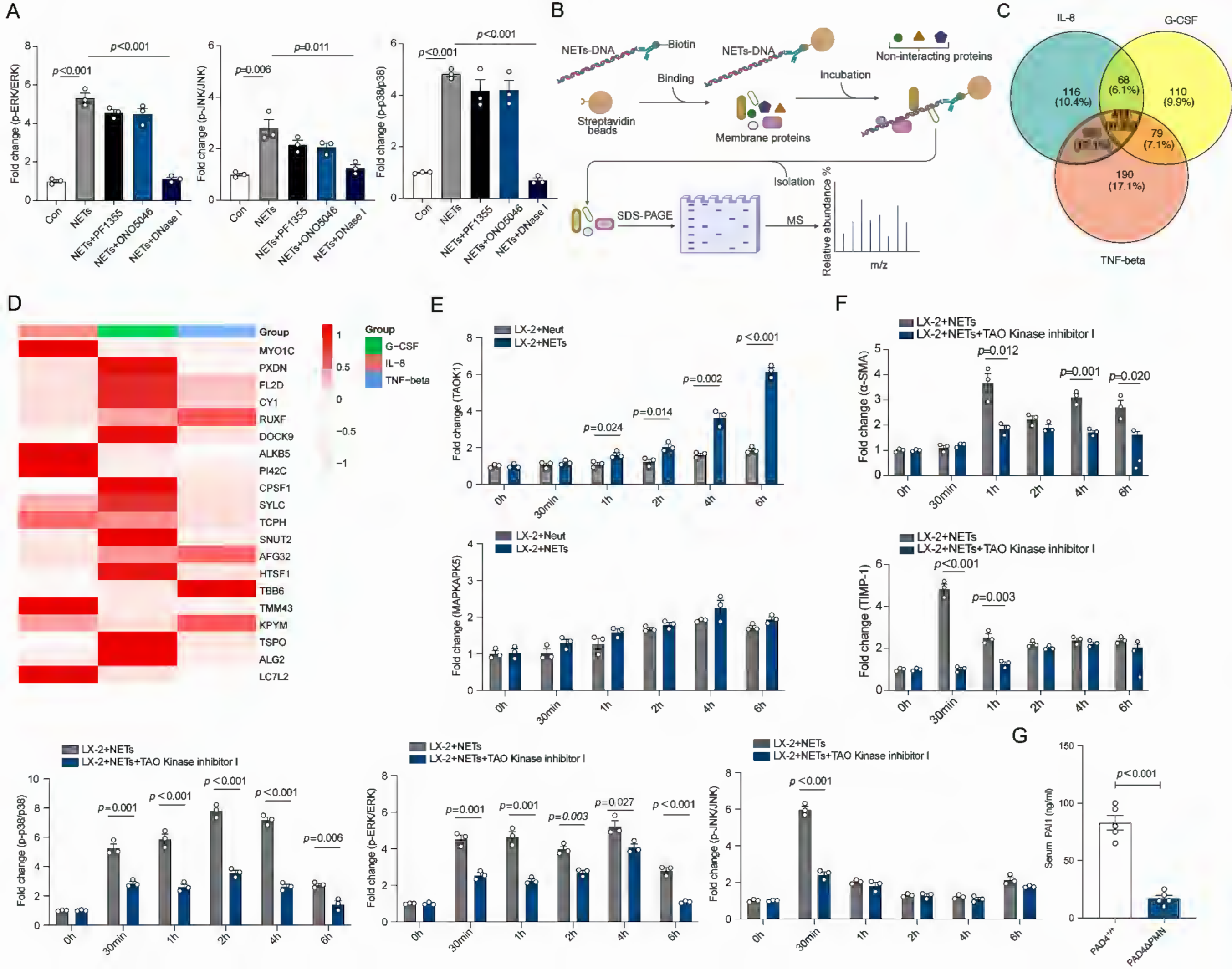

A
B
C
D
E
F
G
NETs-DNA
Biotin
Binding
Streptavidin beads
Membrane proteins
Non-interacting proteins
Incubation
Isolation
SDS-PAGE
MS
Relative abundance %
m/z
IL-8
G-CSF
TNF-beta
Group
MYO1C
PXDN
FL2D
CY1
RUXF
DOCK9
ALKB5
PI42C
CPSF1
SYLC
TCPH
SNUT2
AFG32
HTSF1
TBB6
TMM43
KPYM
TSPO
ALG2
LC7L2
LX-2+Neut
LX-2+NETs
LX-2+NETs+TAO Kinase inhibitor I
Fold change (p-ERK/ERK)
Fold change (p-JNK/JNK)
Fold change (p-p38/p38)
Fold change (TAOK1)
Fold change (MAPKAPK5)
Fold change (α-SMA)
Fold change (TIMP-1)
Serum PAI1 (ng/ml)
Con
NETs
NETs+PF1355
NETs+ONO5046
NETs+DNase I
0h
30min
1h
2h
4h
6h
PAD4+/+
PAD4ΔPMN

**Supplementary Figures**

Supplementary Fig. 1. **Effects of neutrophil depletion on liver under HFD and WD/$CCl_4$ model.** (A) To deplete neutrophils, mice were administered anti-Ly6G antibody intraperitoneally each day during the latter phase of the dietary regimen. The control group received an equivalent dose of IgG2a. (B-C) Flow cytometry was used to analyze the clearance efficiency of neutrophils ($CD11b^+$ $Ly6G^+$populations) in the liver of each group of mice，after intraperitoneal injection of Ly6G antibody. Data are presented as mean ± SEM, based on five independent experiments. Statistical analyses were performed using two-tailed Student Test. Error bars represent mean ± SEM.

Supplementary Fig. 2. **ScRNA-seq analysis revealed differences in the proportions of non-parenchymal cell subsets in MASL and MASH livers.** (A) Uniform manifold approximation and projection (UMAP) analysis of non-parenchymal cells from human MASL (n=6) and MASH (n=6) livers, with each cell colored by its tissue origin. (B) Dot plot illustrating the expression of canonical marker genes across the nine major cell clusters. Dot size indicates cell proportion, while color intensity reflects standardized expression levels. (C) UMAP visualization of transcriptional profiles from 121,020 non-parenchymal cells, partitioned into distinct clusters represented by different colors. (D) Bar plots showing the proportion of non-parenchymal cell subpopulations in human MASL and MASH livers.

Supplementary Fig. 3. **ScRNA-seq reveals neutrophil heterogeneity in human MASL and MASH liver.** (A) Dot plot illustrating the expression of canonical marker genes across the six major cell clusters of myeloid cells. Dot size indicates cell

proportion, while color intensity reflects standardized expression levels. (B) Volcano plot showing differentially expressed genes between neutrophils in MASH and MASL, with significance defined as FDR < 0.01 and |logFC| > 2. (C) Gene Ontology (GO) analysis of downregulated genes in MASH versus MASL, highlighting their associated molecular functions, cellular components, and biological processes. (D) GO analysis of upregulated genes in MASH versus MASL, describing the relevant molecular functions, cellular components, and biological processes.

Supplementary Fig. 4. **Functional enrichment of neutrophil subclusters by single-cell RNA-seq.** (A) Proportion of five neutrophil subpopulations in MASL and MASH groups. (B) Heatmap of significant KEGG pathway terms across neutrophil subtypes (FDR < 0.05). (C) Gene Ontology Biological Process analysis for the Neut-5 subpopulation (FDR < 0.05). (D) Flow cytometry analysis of $PAD4^{+}$ neutrophils from livers of HFD-fed mice.

Supplementary Fig. 5. **Inflammation-related cytokines in liver tissues from MASL and MASH patients.** (A-E) Luminex assay quantification of inflammation-related cytokines in liver tissues from MASL (n=10) and MASH (n=10) patients. Statistically significant cytokines are highlighted. The above cytokines had no statistical difference between the two groups. Statistical analyses were performed using two-tailed Student Test. Error bars represent mean ± SEM.

Supplementary Fig. 6. **Effects of Inhibiting PAD4$^+$ neutrophil and NETs formation on liver under CD, HFD, WD/$CCl_4$ model.** (A) Schematic representation elucidates the construction of PAD4ΔPMN mice model (Created with BioRender.com). (B) Weekly monitored body weight of CD, HFD and WD/$CCl_4$ treated PAD4ΔPMN mice (10 mice/group). (C) qRT-PCR analysis of PAD4 mRNA levels in tissues of 6–8-wk-old male PAD4ΔPMN mice and PAD4$^{+/+}$ mice (n = 5 mice per group). Neutrophils, macrophages, monocytes were isolated from peripheral blood by positive selection using anti-Ly6G, anti-F4/80, anti-Ly6C microbeads, respectively. (D) Neutrophil counts in peripheral blood of PAD4ΔPMN mice and PAD4$^{+/+}$ mice (n = 5 mice per group). （E）Representative immunofluorescence images of H3Cit and MPO staining in livers from WD/$CCl_4$-fed PAD4ΔPMN and PAD4$^{+/+}$ mice. NETs are identified by co-staining with H3Cit, MPO, and DAPI, Scale bars, 20 μm. (F) Representative liver sections showing phenotypic changes, lipid accumulation, and fibrosis, assessed by Masson staining after bone marrow-derived neutrophils (BMDNs) adoptive cell transfer separately. Scale bars, 50 μm. (G) Fibrosis stage of each group. Statistical analyses were performed using two-tailed Student Test. Error bars represent mean ± SEM.

Supplementary Fig. 7. **PAD4$^+$ neutrophils promote hepatic stellate cell activation via NETs formation and MAPK Pathway activation.** (A) Quantitative grayscale analysis of TIMP-1 protein bands from western blots. (B) Quantitative grayscale analysis of COL1A1 protein bands from western blots. (C) Volcano plot showing

differentially expressed genes after NETs stimulated LX-2 cells, with significance defined as FDR < 0.01 and |logFC| > 2. (D) GO analysis of differentially expressed genes, describing the relevant biological processes. (E) Quantitative grayscale analysis of phosphorylated JNK and JNK bands from western blotting. (F) Quantitative grayscale analysis of phosphorylated ERK and ERK bands from western blotting. (G) Quantitative grayscale analysis of phosphorylated p38 and p38 bands from western blots. Statistical analyses were performed using two-tailed Student Test. Error bars represent mean ± SEM.

Supplementary Fig. 8. **Analyzing the role of NETs-DNA and TAOK1 in MAPK pathway activation.** (A) Quantitative grayscale analysis of phosphorylated JNK and JNK bands, phosphorylated ERK and ERK bands, phosphorylated p38 and p38 bands from western blot after adding PF1355, ONO5046, and DNase I. (B) Schematic representation of the NETs-DNA pull-down assay methodology used to identify proteins interacting with NETs-DNA. (C) LC/MS was used to detect LX-2 cell membrane proteins obtained by DNA pull down analysis, depicted in a Venn diagram. (D) A heatmap displays the top 20 proteins identified through DNA pull-down assays. (E) Quantitative grayscale analysis of TAOK1 and MAPKAPK5 bands from western blotting. (F) Quantitative grayscale analysis of a-SMA, TIMP-1, phosphorylated JNK and JNK bands, phosphorylated ERK and ERK bands, phosphorylated p38 and p38 bands from western blot after adding TAO Kinase inhibitor I. (G) Serum PAI-1 levels (ng/mL) measured by ELISA in WD/$CCl_4$-fed $PAD4^{+/+}$ and PAD4ΔPMN mice.

Statistical analyses were performed using one-way ANOVA and two-tailed Student Test. Error bars represent mean ± SEM.

**Supplementary Table 1. Characteristics of subjects whose Liver biopsy tissues were analyzed.**

| Variables | **non-MASLD** (n=63) | **non-MASH** (n=77) | **MASH** (n=125) | **p Value** |
|---|---|---|---|---|
| **Demographic parameters** | | | | |
| Age (years) | 35 (27, 44) | 31 (26, 38) | 30 (25, 37) | 0.021 |
| Female (n, %) | 53, 84.13 | 56, 72.73 | 87, 69.60 | 0.097 |
| BMI (kg/m2) | 35.50 (32.20, 40.70) | 36 (32.15, 42.55) | 38.50 (34.75, 44.81) | 0.007 |
| **Metabolic parameters** | | | | |
| ALT (U/L) | 22.50 (17.30, 29.50) | 30.00 (20.40, 49.50) | 49.90 (32.95, 73.65) | 0.000 |
| AST (U/L) | 19.70 (17.30, 25.80) | 24.50 (18.90, 34.20) | 30.80 (23.60, 41.70) | 0.000 |
| GGT (U/L) | 25.40 (18.50, 51.70) | 34.10 (23.00, 56.85) | 40.80 (27.70, 60.20) | 0.001 |
| TbiL (U/L) | 10.00 (7.20, 12.90) | 10.70 (7.45, 12.30) | 9.90 (7.50, 12.60) | 0.969 |
| ALB(g/L) | 40.07±3.44 | 40.81±3.66 | 40.78±4.05 | 0.420 |
| TG (mmol/L) | 4.62 (4.14, 5.27) | 4.99 (4.17, 5.77) | 4.90 (4.18, 5.61) | 0.379 |
| TC (mmol/L) | 1.26 (0.98, 1.76) | 1.64 (1.26, 2.26) | 1.57 (1.18, 2.30) | 0.001 |
| HDL-C (mmol/L) | 1.15 (0.92, 1.32) | 1.02 (0.87, 1.23) | 1.04 (0.92, 1.18) | 0.059 |
| LDL-C (mmol/L) | 2.93 (2.55, 3.35) | 3.16 (2.58, 3.82) | 3.10 (2.60, 3.61) | 0.256 |
| **Liver histopathologic characteristics** | | | | |
| Neutrophil count (n, per high-power field) | 2 (1, 4) | 5 (3, 11) | 7 (5, 12) | 0.000 |

**Supplementary Table 2. Liver histopathologic characteristics of the study individuals**

| Liver histopathologic characteristics | Total patients (n=265) |
|---|---|
| **Steatosis, n (%)** | |
| 0 | 63 (23.8) |
| 1 | 94 (35.5) |
| 2 | 75 (28.3) |
| 3 | 33 (12.5) |
| **Ballooning, n (%)** | |
| 0 | 19 (7.2) |
| 1 | 96 (36.2) |
| 2 | 150 (56.6) |
| **Lobular inflammation, n (%)** | |
| 0 | 27 (10.2) |
| 1 | 94 (35.5) |
| 2 | 127 (47.9) |
| 3 | 17 (6.4) |
| **Fibrosis, n (%)** | |
| 0 | 24 (9.1) |
| 1 | 93 (35.1) |
| 2 | 86 (32.5) |
| 3 | 59 (22.3) |
| 4 | 3 (1.1) |
| **NAS, n (%)** | |
| <3 | 40 (15.09) |
| 3-4 | 98 (36.98) |
| >4 | 127 (47.92) |

**Supplementary Table 3. Absolute liver tissue cytokine concentrations(pg/ml) by group.**

| cytokine | | MASH1 | MASH2 | MASH3 | MASH4 | MASH5 | MASH6 | MASH7 | MASH8 | MASH9 | MASH10 | | MASL1 | MASL2 | MASL3 | MASL4 | MASL5 | MASL6 | MASL7 | MASL8 | MASL9 | MASL10 |
|---|---|---|---|---|---|---|---|---|---|---|---|---|---|---|---|---|---|---|---|---|---|---|
| IL-2Ralpha | | 30.92 | 27.20 | 34.15 | 33.92 | 26.97 | 40.55 | 42.37 | 35.07 | 33.69 | 31.85 | | 37.36 | 24.86 | 22.99 | 36.91 | 33.69 | 26.27 | 29.53 | 28.14 | 31.85 | 27.44 |
| MIG | | 977.60 | 2429.00 | 916.87 | 793.81 | 525.52 | 2161.00 | 4377.00 | 1469.00 | 1587.00 | 1109.00 | | 420.75 | 507.84 | 1283.00 | 367.84 | 575.95 | 633.05 | 355.39 | 334.70 | 1976.00 | 302.15 |
| MIP-1beta | | 559.56 | 251.22 | 169.11 | 228.30 | 300.54 | 233.97 | 224.36 | 129.52 | 114.18 | 130.69 | | 149.04 | 121.01 | 115.16 | 101.33 | 77.57 | 134.92 | 83.07 | 179.43 | 242.84 | 118.47 |
| IL-6 | | 21.94 | 6.00 | 8.72 | 16.65 | 7.45 | 9.60 | 7.19 | 6.00 | 7.99 | 7.85 | | 14.00 | 11.89 | 3.70 | 10.34 | 5.87 | 4.20 | 3.95 | 33.31 | 6.79 | 7.05 |
| IFN-alpha2 | | 31.51 | 19.44 | 23.13 | 27.59 | 25.75 | 36.81 | 38.14 | 28.67 | 33.25 | 31.86 | | 33.59 | 27.59 | 22.36 | 43.00 | 33.76 | 28.85 | 26.12 | 35.47 | 25.38 | 36.48 |
| IFN-gamma | | 148.40 | 183.14 | 184.50 | 186.99 | 155.31 | 205.71 | 211.09 | 203.46 | 216.47 | 183.14 | | 194.67 | 188.12 | 171.33 | 213.34 | 191.51 | 178.38 | 184.28 | 174.51 | 181.33 | 186.77 |
| SDF-1alpha | | 229.56 | 562.75 | 209.72 | 214.56 | 448.64 | 444.62 | 249.57 | 331.83 | 364.45 | 476.06 | | 353.65 | 222.78 | 269.14 | 274.29 | 267.20 | 294.00 | 153.98 | 261.36 | 424.42 | 307.16 |
| IL-1ra | | 9255.00 | 19837.00 | 18856.00 | 18128.00 | 13379.00 | 20887.00 | 18320.00 | 19932.00 | 20508.00 | 13146.00 | | 18358.00 | 16946.00 | 12869.00 | 15199.00 | 16703.00 | 17262.00 | 21224.00 | 11108.00 | 15593.00 | 13950.00 |
| MC | | 4.8 | 5.34 | 5.12 | 5.79 | 4.67 | 6.67 | 6.45 | 5.79 | 5.34 | 5.90 | | 5.90 | 4.21 | 4.67 | 6.45 | 4.44 | 4.89 | 4.44 | 3.76 | 5.79 | 5.12 |

| | | | | | | | | | | | | | | | | | | | | | | |
|---|---|---|---|---|---|---|---|---|---|---|---|---|---|---|---|---|---|---|---|---|---|---|
| P-3 | | 9 | | | | | | | | | | | | | | | | | | | | |
| IL-16 | | 2015.00 | 2555.00 | 1944.00 | 3050.00 | 1993.00 | 2732.00 | 2757.00 | 2862.00 | 2165.00 | 1769.00 | | 3091.00 | 2483.00 | 2536.00 | 1754.00 | 1692.00 | 2071.00 | 2051.00 | 1922.00 | 2463.00 | 1157.00 |
| IL-12p40 | | 213.56 | 377.07 | 332.95 | 264.56 | 189.49 | 315.19 | 315.19 | 332.95 | 344.75 | 344.75 | | 285.46 | 183.47 | 243.60 | 294.40 | 243.60 | 207.54 | 201.53 | 183.47 | 261.57 | 177.45 |
| LIF | | 69.31 | 77.88 | 85.35 | 94.89 | 54.25 | 73.60 | 94.89 | 77.88 | 71.46 | 65.02 | | 80.02 | 58.56 | 56.40 | 80.02 | 66.09 | 54.25 | 62.87 | 51.01 | 75.74 | 49.93 |
| TNF-beta | | 532.94 | 403.15 | 386.10 | 340.05 | 462.35 | 402.90 | 347.42 | 349.63 | 255.72 | 323.40 | | 353.33 | 232.73 | 303.46 | 287.97 | 170.04 | 421.86 | 222.69 | 385.10 | 445.34 | 331.71 |
| IL-5 | | 134.14 | 138.86 | 181.71 | 142.41 | 160.21 | 118.90 | 214.15 | 223.79 | 216.56 | 170.94 | | 182.91 | 175.73 | 150.70 | 268.46 | 279.33 | 186.51 | 147.14 | 187.71 | 161.40 | 231.03 |
| GM-CSF | | 17.02 | 16.21 | 20.15 | 19.99 | 18.41 | 20.64 | 27.45 | 18.24 | 27.25 | 21.90 | | 22.19 | 20.85 | 18.91 | 24.58 | 37.50 | 20.44 | 18.91 | 20.64 | 21.01 | 23.91 |
| TNF-alpha | | 55.21 | 44.50 | 49.89 | 48.10 | 45.58 | 58.02 | 59.07 | 49.53 | 52.38 | 50.24 | | 52.38 | 45.94 | 38.68 | 57.32 | 48.10 | 44.86 | 38.31 | 47.38 | 46.66 | 52.73 |
| RANTES | | 5871.00 | 1867.00 | 1285.00 | 1026.00 | 2090.00 | 1207.00 | 1013.00 | 950.18 | 593.09 | 814.86 | | 1159.00 | 538.49 | 805.94 | 647.43 | 349.79 | 1195.00 | 507.11 | 1188.00 | 1775.00 | 756.12 |
| IL-2 | | 8.94 | 9.53 | 10.33 | 11.74 | 8.75 | 11.54 | 13.58 | 11.54 | 11.54 | 11.14 | | 12.76 | 7.58 | 7.96 | 13.58 | 9.93 | 8.94 | 10.33 | 8.75 | 9.73 | 10.73 |
| IL-1beta | | 11.81 | 11.48 | 12.49 | 14.57 | 11.90 | 12.95 | 14.74 | 11.98 | 13.41 | 12.32 | | 13.91 | 10.97 | 10.21 | 15.90 | 11.94 | 10.72 | 11.98 | 12.82 | 12.65 | 11.65 |
| IL-18 | | 99.55 | 627.36 | 189.79 | 208.68 | 101.92 | 156.77 | 168.24 | 147.19 | 150.50 | 137.99 | | 199.88 | 225.24 | 152.35 | 195.20 | 140.93 | 179.18 | 147.19 | 151.42 | 338.62 | 124.41 |
| Eot | | 10. | 19.69 | 10.3 | 10.5 | 14.1 | 19.4 | 17.7 | 15.7 | 17.8 | 19.4 | | 15.4 | 12.6 | 15.0 | 16.9 | 14.2 | 17.4 | 10.5 | 15.5 | 16.4 | 16.0 |

| | | | | | | | | | | | | | | | | | | | | | | |
|---|---|---|---|---|---|---|---|---|---|---|---|---|---|---|---|---|---|---|---|---|---|---|
| axin | | 94 | | 1 | 8 | 3 | 9 | 0 | 4 | 0 | 4 | | 8 | 7 | 1 | 8 | 9 | 9 | 2 | 3 | 6 | 0 |
| Basic FGF | | 262.73 | 180.27 | 441.11 | 524.47 | 191.99 | 102.93 | 567.07 | 433.06 | 185.33 | 171.16 | | 433.68 | 336.31 | 277.50 | 408.32 | 374.72 | 306.32 | 442.35 | 190.08 | 464.61 | 118.67 |
| VEGF | | 155.14 | 183.57 | 197.17 | 215.02 | 172.43 | 213.75 | 209.95 | 231.95 | 242.47 | 242.47 | | 248.00 | 188.13 | 147.06 | 308.28 | 186.83 | 207.41 | 186.83 | 213.75 | 211.22 | 221.32 |
| beta-NGF | | 25.26 | 18.12 | 26.03 | 29.90 | 25.77 | 33.49 | 39.09 | 28.87 | 37.44 | 28.36 | | 37.06 | 29.13 | 14.88 | 45.14 | 32.47 | 27.45 | 22.80 | 37.06 | 26.81 | 37.06 |
| PDGF-BB | | 286.34 | 238.84 | 175.48 | 219.26 | 190.84 | 303.49 | 312.04 | 299.21 | 339.72 | 337.60 | | 288.49 | 219.26 | 247.51 | 358.79 | 260.49 | 294.92 | 190.84 | 249.68 | 260.49 | 337.60 |
| IP-10 | | 803.19 | 2835.00 | 490.25 | 1213.00 | 786.21 | 2194.00 | 2186.00 | 1492.00 | 1046.00 | 897.96 | | 606.10 | 834.22 | 1107.00 | 180.59 | 601.89 | 613.63 | 235.50 | 259.04 | 1192.00 | 248.99 |
| IL-13 | | 7.65 | 4.77 | 5.18 | 6.25 | 7.10 | 9.39 | 8.55 | 7.25 | 10.33 | 9.15 | | 8.95 | 6.65 | 5.33 | 10.33 | 8.00 | 7.45 | 5.33 | 8.85 | 5.79 | 11.84 |
| IL-4 | | 3.66 | 3.46 | 3.26 | 3.79 | 3.19 | 4.05 | 4.37 | 4.05 | 3.59 | 3.56 | | 3.88 | 3.16 | 2.99 | 4.30 | 3.49 | 2.92 | 3.39 | 3.12 | 3.52 | 2.99 |
| MCP-1 | | 95.48 | 79.39 | 56.72 | 140.79 | 51.04 | 67.78 | 60.06 | 54.09 | 60.67 | 53.46 | | 41.84 | 57.59 | 42.11 | 71.55 | 55.10 | 39.66 | 38.84 | 180.61 | 52.44 | 57.10 |
| IL-8 | | 59.01 | 39.72 | 37.85 | 33.95 | 28.90 | 26.25 | 11.70 | 20.06 | 11.61 | 11.61 | | 11.35 | 10.56 | 10.04 | 9.69 | 8.83 | 8.48 | 6.08 | 26.07 | 16.00 | 12.31 |
| MIP-1alpha | | 144.46 | 45.01 | 45.88 | 67.44 | 53.90 | 68.60 | 43.69 | 19.74 | 37.41 | 17.06 | | 24.64 | 26.22 | 12.48 | 20.68 | 18.84 | 13.42 | 8.74 | 45.39 | 37.05 | 22.80 |
| IL-10 | | 26.77 | 19.64 | 30.94 | 37.49 | 28.56 | 56.12 | 48.16 | 41.95 | 50.82 | 50.52 | | 36.90 | 35.11 | 32.14 | 54.94 | 54.06 | 35.71 | 24.99 | 46.98 | 27.97 | 48.16 |
| G-CS | | 3266.0 | 1362.00 | 1357.00 | 1915.00 | 1567.00 | 1919.00 | 1385.00 | 985.65 | 1376.00 | 858.87 | | 1160.00 | 1072.00 | 617.89 | 1072.00 | 1127.00 | 784.86 | 588.06 | 1375.00 | 1200.00 | 1104.00 |

| | | | | | | | | | | | | | | | | | | | | | | |
|---|---|---|---|---|---|---|---|---|---|---|---|---|---|---|---|---|---|---|---|---|---|---|
| F | | 0 | | | | | | | | | | | | | | | | | | | | |
| GRO-alpha | | 891.96 | 711.40 | 826.88 | 853.52 | 812.76 | 800.86 | 881.89 | 839.69 | 698.36 | 753.30 | | 870.61 | 673.11 | 660.92 | 886.37 | 666.36 | 739.51 | 764.45 | 715.28 | 842.00 | 698.36 |
| HGF | | 2035.00 | 4662.00 | 3798.00 | 2626.00 | 1846.00 | 3048.00 | 2415.00 | 3405.00 | 3613.00 | 3202.00 | | 2716.00 | 1696.00 | 2560.00 | 2448.00 | 2258.00 | 2009.00 | 1471.00 | 1598.00 | 2997.00 | 1087.00 |
| IL-1alpha | | 49.40 | 101.05 | 153.15 | 109.19 | 63.77 | 154.77 | 235.70 | 172.63 | 198.56 | 107.56 | | 174.26 | 161.27 | 135.25 | 214.73 | 237.32 | 112.45 | 159.65 | 111.64 | 140.13 | 127.11 |
| IL-3 | | 2.03 | 1.76 | 1.76 | 2.22 | 1.40 | 1.94 | 2.36 | 1.67 | 2.12 | 1.49 | | 1.85 | 1.31 | 1.31 | 2.17 | 1.76 | 1.40 | 1.76 | 1.67 | 1.80 | 1.22 |
| SCF | | 57.56 | 59.15 | 81.81 | 86.55 | 51.17 | 60.35 | 81.41 | 62.34 | 51.57 | 79.03 | | 71.89 | 57.16 | 51.17 | 110.54 | 59.95 | 58.36 | 75.86 | 55.56 | 71.89 | 46.38 |
| TRAIL | | 126.73 | 178.00 | 107.76 | 147.75 | 203.47 | 178.78 | 137.88 | 112.03 | 149.72 | 124.73 | | 159.23 | 84.59 | 128.93 | 171.70 | 92.33 | 195.00 | 134.21 | 133.51 | 147.56 | 208.28 |
| M-CSF | | 20.05 | 61.08 | 41.23 | 40.77 | 22.14 | 32.74 | 33.81 | 43.18 | 33.12 | 39.72 | | 43.33 | 34.87 | 31.83 | 40.02 | 34.57 | 24.39 | 32.74 | 26.01 | 35.25 | 23.61 |
| CTACK | | 55.33 | 75.58 | 63.63 | 68.77 | 53.58 | 86.03 | 89.38 | 80.65 | 92.05 | 110.28 | | 78.29 | 61.56 | 80.65 | 102.02 | 87.04 | 69.80 | 66.38 | 77.27 | 73.20 | 71.16 |
| IL-15 | | 512.08 | 285.36 | 402.31 | 466.42 | 349.32 | 564.70 | 637.35 | 520.26 | 580.62 | 428.23 | | 544.62 | 445.30 | 238.17 | 617.96 | 662.31 | 406.66 | 371.59 | 458.00 | 445.30 | 578.64 |
| IL-7 | | 35.36 | 37.65 | 43.07 | 42.63 | 48.81 | 59.71 | 60.76 | 51.63 | 59.92 | 64.70 | | 62.63 | 39.93 | 44.19 | 84.44 | 58.66 | 59.08 | 36.28 | 54.00 | 48.38 | 64.49 |
| IL-12p70 | | 4.63 | 3.89 | 4.89 | 4.63 | 4.38 | 6.20 | 7.56 | 6.06 | 6.73 | 6.46 | | 6.46 | 6.20 | 4.26 | 8.11 | 7.83 | 5.15 | 4.63 | 6.46 | 4.76 | 7.83 |
| IL-17 | | 24.55 | 29.05 | 32.44 | 35.07 | 29.05 | 39.98 | 43.00 | 36.21 | 46.22 | 39.22 | | 41.11 | 30.55 | 27.92 | 62.29 | 35.07 | 38.47 | 32.25 | 43.00 | 36.96 | 51.13 |
| IL- | | 293 | 257.0 | 245. | 218. | 268. | 259. | 253. | 232. | 203. | 228. | | 253. | 173. | 210. | 227. | 148. | 248. | 167. | 243. | 258. | 241. |

| | | | | | | | | | | | | | | | | | | | | | |
|---|---|---|---|---|---|---|---|---|---|---|---|---|---|---|---|---|---|---|---|---|---|
| 9 | | .38 | 2 | 85 | 97 | 20 | 81 | 88 | 59 | 59 | 40 | | 18 | 74 | 93 | 70 | 22 | 64 | 38 | 06 | 07 | 32 |
| SC GF-beta | | 4060.00 | 4500.00 | 5375.00 | 5926.00 | 4638.00 | 5099.00 | 5628.00 | 4661.00 | 6338.00 | 5582.00 | | 5846.00 | 4938.00 | 3828.00 | 6636.00 | 4476.00 | 5111.00 | 4984.00 | 6132.00 | 5582.00 | 6270.00 |

**Supplementary Table 4. Key Resources (reagents)**

| REAGENT | SOURCE | Catalog # | RRID |
|---|---|---|---|
| rabbit monoclonal anti-MPO antibody | Abcam | ab208670 | AB_2864724 |
| rabbit monoclonal anti-αSMA antibody | Abcam | ab124964 | AB_11129103 |
| rabbit monoclonal anti-TIPM-1 antibody | Abcam | ab211926 | AB_3095674 |
| rabbit monoclonal anti-COL1A1 antibody | Abcam | ab138492 | AB_2861258 |
| rabbit monoclonal anti-RAS antibody | Cell Signaling Technology | 91054 | AB_3697442 |
| rabbit monoclonal anti-bRAF antibody | Cell Signaling Technology | 9433 | AB_2259354 |
| rabbit monoclonal anti- phospho-bRAF antibody | Cell Signaling Technology | 2696 | AB_390721 |
| rabbit monoclonal anti-cRAF antibody | Cell Signaling Technology | 12552 | AB_2728706 |
| rabbit monoclonal anti- phospho-cRAF antibody | Cell Signaling Technology | 9421 | AB_330759 |
| rabbit monoclonal anti-MEK antibody | Cell Signaling Technology | 8727 | AB_10829473 |
| rabbit monoclonal anti- phospho-MEK antibody | Cell Signaling Technology | 9154 | AB_2138017 |
| rabbit monoclonal anti-JNK antibody | Cell Signaling Technology | 9252 | AB_2250373 |
| rabbit monoclonal anti- phospho-JNK antibody | Cell Signaling Technology | 4668 | AB_823588 |
| rabbit monoclonal anti-ERK antibody | Cell Signaling Technology | 4695 | AB_390779 |
| rabbit monoclonal anti- phospho-ERK antibody | Cell Signaling Technology | 4370 | AB_2315112 |
| rabbit monoclonal anti-p38 antibody | Cell Signaling Technology | 8690 | AB_10999090 |
| rabbit monoclonal anti- phospho-p38 antibody | Cell Signaling Technology | 4511 | AB_2139682 |
| rabbit monoclonal anti-GAPDH antibody | Cell Signaling Technology | 2118 | AB_561053 |
| mouse monoclonal anti-GAPDH antibody | Cell Signaling Technology | 97166 | AB_2756824 |

| | | | |
|---|---|---|---|
| rabbit monoclonal anti-TAOK1 antibody | Thermo Fisher Scientific | PA5-101868 | AB_2851300 |
| Mouse monoclonal anti-MAPKAPK5 antibody | Thermo Fisher Scientific | H00008550-M02 | AB_10718167 |
| mouse monoclonal anti-MPO antibody | Abcam | ab90810 | AB_2146325 |
| rabbit monoclonal anti-H3cit antibody | Abcam | ab5103 | AB_304752 |
| Rhodamine Red-X (RRX) goat anti-mouse IgG (H+L) | Jackson | 115-295-146 | AB_2338766 |
| FITC-AffiniPure goat anti-rabbit IgG (H+L) | Jackson | 111-095-003 | AB_2337972 |
| APC Cyanine7conjugated anti-mouse CD45(clone 30F11) | Biolegend | 103115 | AB_312980 |
| PEvio770 conjugate anti-mouse CD11b (clone M1/70) | Thermo Fisher Scientific | 12-0112-82 | AB_2734869 |
| APC conjugated anti-mouse F4/80 (clone BM8) | Thermo Fisher Scientific | 14-4801-82 | AB_467558 |
| FITC-conjugated anti-mouse Ly6C (clone AL21) | BD Biosciences | 553104 | AB_394628 |
| PE-conjugated anti-mouse Ly6G (clone) | Thermo Fisher Scientific | 12-9668-82 | AB_2572720 |
| Alexa Fluor® 488 monoclonal anti-rabbit PAD4 | Abcam | ab321855 | AB_3714924 |
| APC conjugated anti-mouse CD45 (clone I3/2.3) | Biolegend | 147707 | AB_2563539 |
| PE-conjugated anti-mouse Ly6G (clone 1A8) | Biolegend | 127607 | AB_1186104 |
| PerCP/Cyanine5.5 anti-mouse CD11b (M1/70) | Biolegend | 101227 | AB_893233 |
| IL-8 | Sigma-Aldrich | I1645 | N/A |
| G-CSF | Sigma-Aldrich | SRP3263 | N/A |
| TNF-beta | Sigma-Aldrich | T7799 | N/A |
| Myeloperoxidase Inhibitor PF1355 | Cayman Chemical | 1435467-38-1 | N/A |
| ELANE Inhibitor | Cayman Chemical | 201677-61-4 | N/A |

| | | | |
|---|---|---|---|
| ONO5046 | | | |
| DNase I | Sigma-Aldrich | AMPD1 | N/A |
| TAO Kinase inhibitor 1 | MedChemexpress | HY-112136 | N/A |
| MAPK Inhibitor U0126 | Sigma-Aldrich | 1173097-76-1 | N/A |
