# Supplementary material for: PAD4+ neutrophils promote hepatic stellate cell activation and accelerate MASH fibrosis progression viaNET-DNA/TAOK1/MAPK pathways
Source: JCI Insight. 2026 Jan 9;11(1):e191479. doi: 10.1172/jci.insight.191479 (PMC12890522; doi:10.1172/jci.insight.191479)

Figure 5A

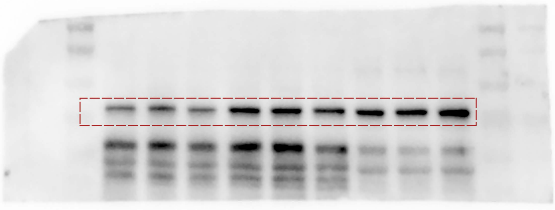

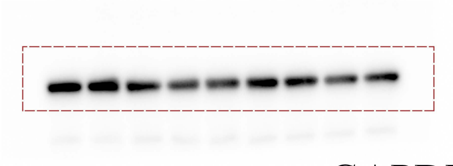

Figure 5E

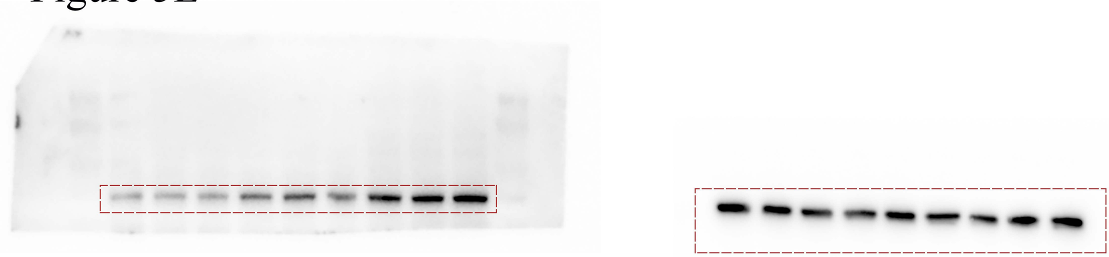

PAD4 GAPDH

Figure 5K

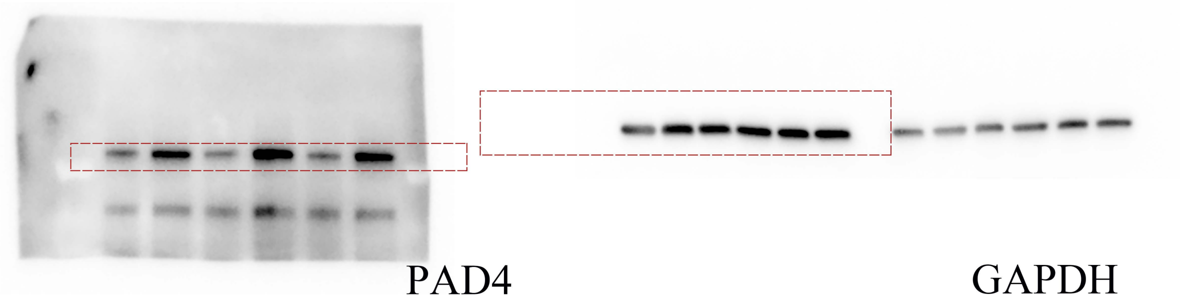

Figure 7E

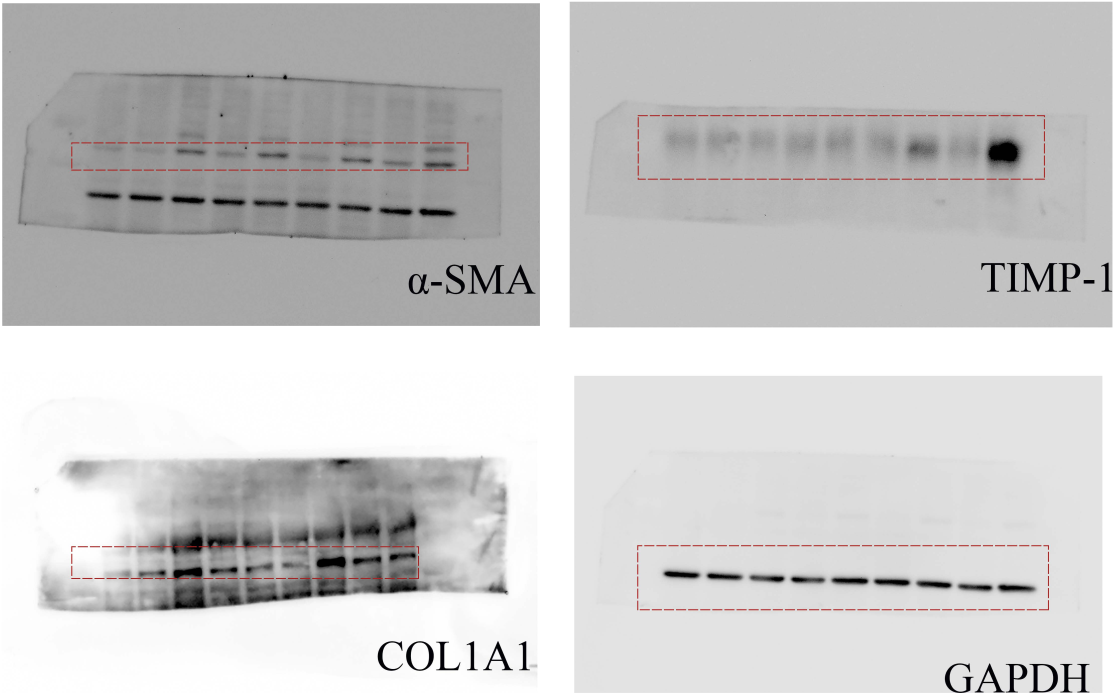

Figure 7I

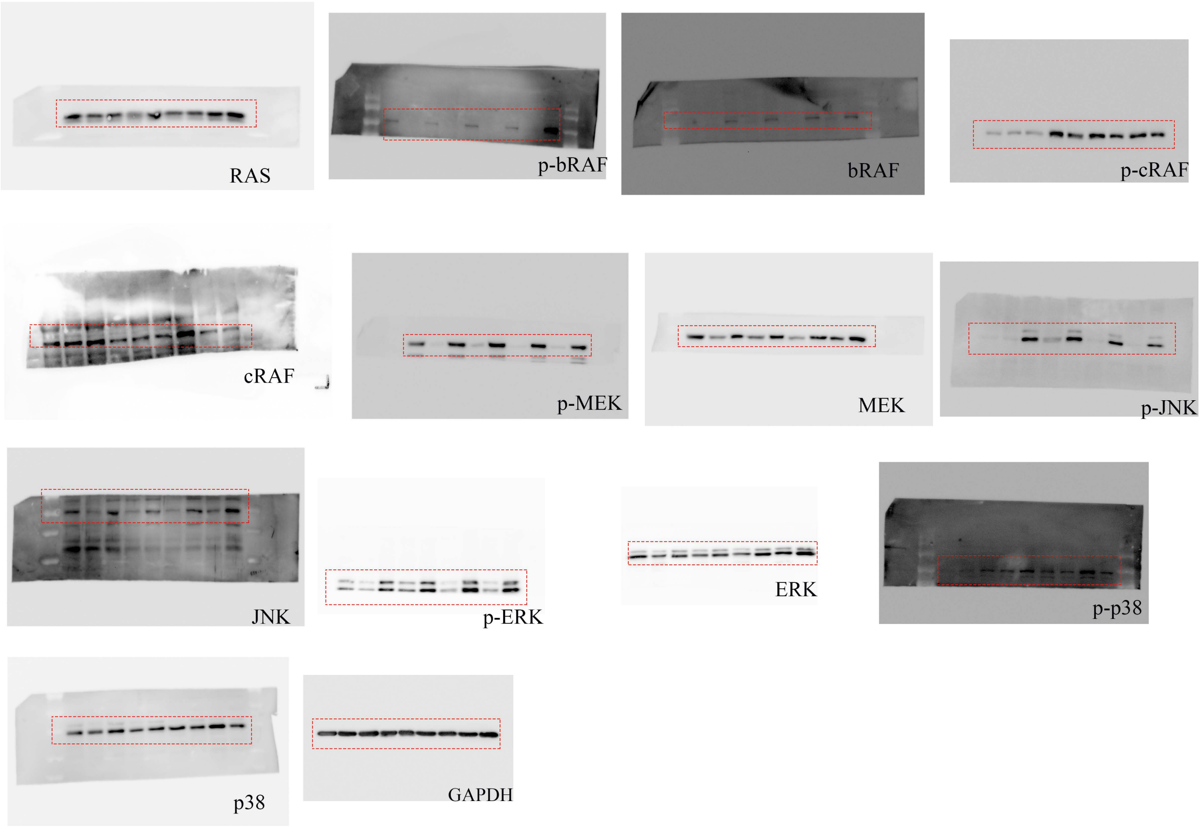
RAS
p-bRAF
bRAF
p-cRAF
cRAF
p-MEK
MEK
p-JNK
JNK
p-ERK
ERK
p-p38
p38
GAPDH

Figure 8C

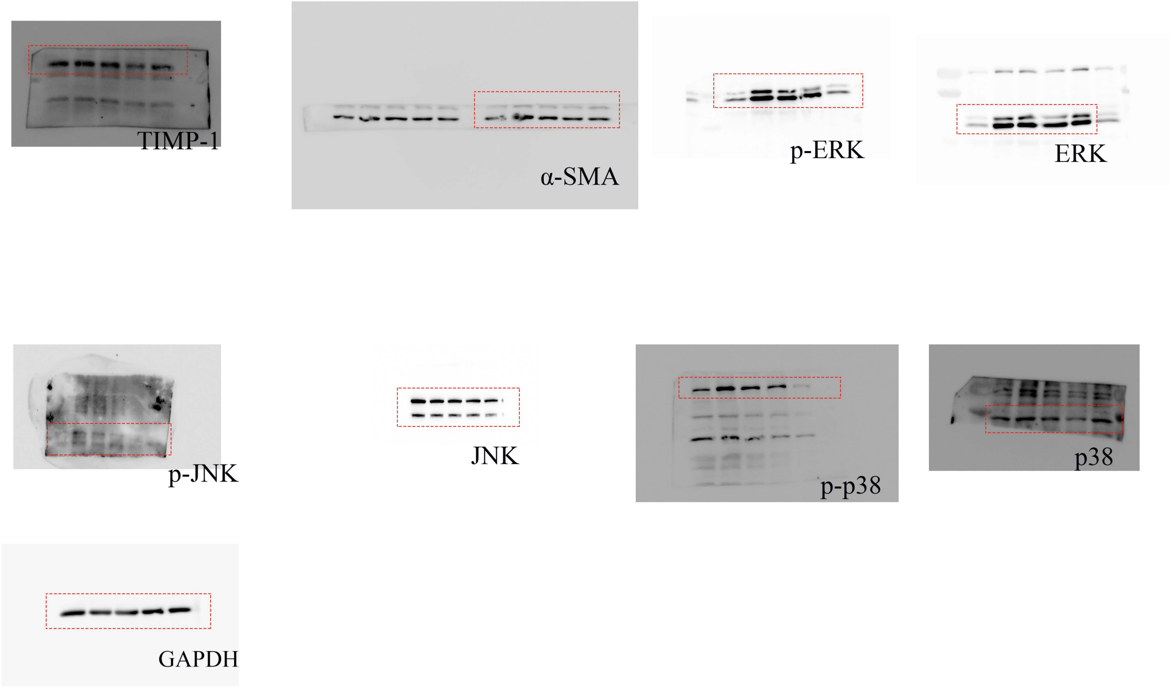

Figure 8G

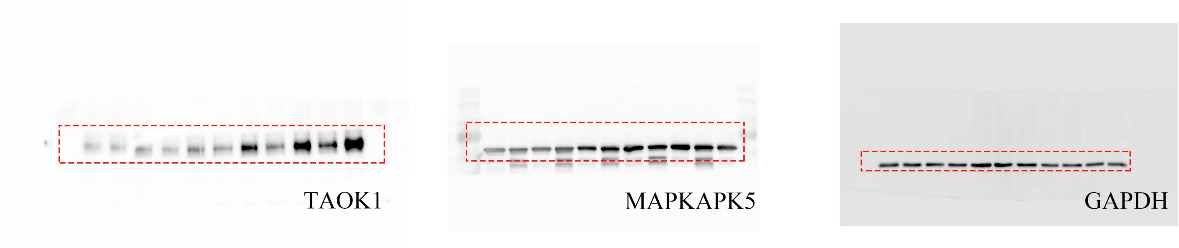

Figure 8J

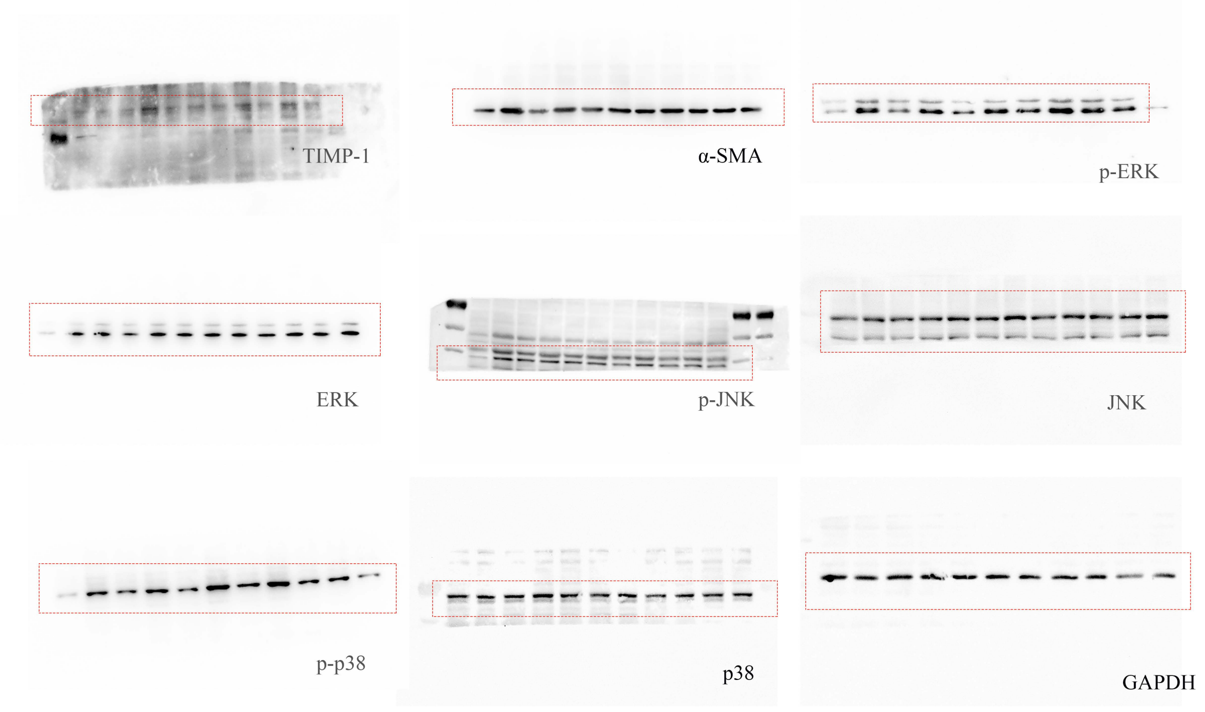

Supplement: Unedited blot and gel images [file jciinsight-11-191479-s064.pdf]
